# Marvelous Characteristics of Hydroxyl-Functionalized Azo-Dye Polymers for Electrocatalytic Sensing: An Informative Review

**DOI:** 10.1021/acspolymersau.5c00145

**Published:** 2025-11-24

**Authors:** Lokman Liv

**Affiliations:** Electrochemistry Laboratory, Chemistry Group, The Scientific and Technological Research Council of Turkey, National Metrology Institute, TUBITAK UME, Gebze, Kocaeli 41470, Turkey

**Keywords:** hydroxyl-functionalized azo-dye, electropolymerization, electrocatalytic determination, electrochemical sensors, electrochemical sensing mechanism, extended Hückel method, molecular mechanics force field method, cyclic voltammetry

## Abstract

Hydroxyl-functionalized azo-dye monomers can be electropolymerized onto a supporting electrode surface, providing an electrocatalytic sensing feature due to their unique physicochemical and electrochemical properties. These polymers combine the versatile redox activity of azo groups with the improved conductivity and stability yielded by the conjugation of aromatic functional groups and hydroxyl functionalities. These properties synergistically enable the polymer film platforms to determine target analytes with greater accuracy, selectivity, and sensitivity than bare platforms, thereby facilitating electrocatalytic detection. Moreover, these polymer films have exhibited exceptional stability, consistent reproducibility, and remarkable resistance to fouling, making them well-suited for practical applications. This study evaluated the detection performance of platforms produced by the electropolymerization of hydroxyl-containing azo-dye monomers, and the underlying reasons for the observed electrocatalytic activity of these polymers were discussed using the extended Hückel charge and the Molecular Mechanics Force Field (MM2) calculations. Consequently, this review highlights the potential of hydroxyl-containing azo polymer-based electrodes as advanced electrochemical sensing platforms and provides excellent foresight in their use.

## Graphical abstract

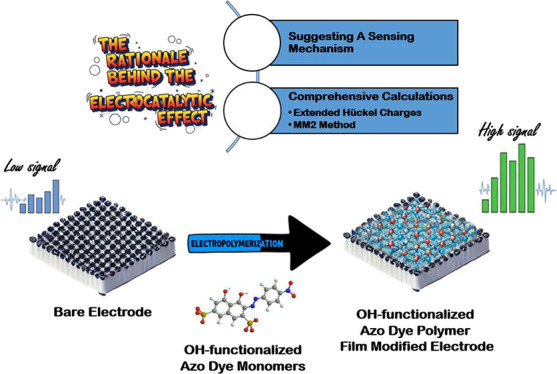

## 1 Introduction

Azo dyes are synthetic organic compounds characterized by one or more azo (—N=N) functional groups. These functional groups typically conjugate with aromatic structures, acting as chromophores. Azo dyes are commonly synthesized from phenols and aromatic amines, accounting for nearly 70% of the dyes produced globally. Their extensive functionalization capabilities and a broad spectrum of color diversity have led to widespread use in industries such as food, pharmaceuticals, cosmetics, and textiles. The conjugated system formed by the azo bond causes bathochromic shifts, resulting in hues ranging from yellow to red, and even enables blue shifts under specific conditions. Aromatic substitutions influence color properties and allow for extensive structural modifications, providing precise control over the physical and chemical properties. This adaptability contributes to increased thermal and chemical stability.^1–3^

When hydroxyl groups are used for functionalization, they enhance or introduce properties such as hydrogen bonding, increased electron density, additional resonance structures, reactivity, and improved solubility. These characteristics open new avenues for hydroxyl-functionalized azo dyes in various fields including biomedical applications (dyes, drug delivery vehicles, and imaging probes), the textile industry (production of color-stable products), environmental applications (agents for pollutant removal), energy storage (components in batteries and supercapacitors), catalysis (enhancing photocatalytic reactivity) and optical and electrochemical sensing (probes for sensor applications).^4–8^

Among these applications, using hydroxyl-functionalized azo dye polymers as electrochemical sensor platforms has recently gained significant popularity.^4,9–12^ Their low cost, pronounced electrocatalytic activity, ease of stable and reproducible electropolymerization, and the inherent advantages of electrochemical methods—such as simplicity, affordability, portability, high accuracy, sensitivity, and selectivity—make them beneficial materials for sensor development.^13–15^ In addition, it is generally well established that bare electrodes exhibit high impedance and slow redox kinetics.^9,16^ In contrast, when modified with polymer films, these electrodes demonstrate antifouling properties, particularly beneficial in analyses involving biological matrices such as blood, urine, or saliva. The polymer coating effectively minimizes biofouling caused by lipids, proteins, and cellular materials. Consequently, the signal-to-noise ratio is significantly improved, leading to enhanced analytical sensitivity.^17–19^ Owing to their antifouling characteristics, the stability of these polymer-modified electrodes is also markedly improved. A review of studies on polymer-based electrodes reveals that a poly­(tartrazine)-modified electrode retained 92.8% of its initial response after 12 days for uric acid detection,^10^ while a poly­(ponceau)-modified electrode preserved 91.6% stability after 14 days for levodopa determination.^11^ Similarly, a poly­(azorubin S)-modified electrode maintained 92.8% of its response after 14 days during nicotine detection.^15^ Furthermore, the relative standard deviation values for repeatability were consistently below 5%, and the reproducibility values were below 8% for all these platforms. The results collectively confirm that the developed electrochemical sensing platforms exhibit excellent long-term stability and measurement reliability. Regarding other physicochemical properties of these polymer films, it has been reported that certain specific azo-based polymer films exhibit thicknesses ranging from one to several micrometers after electrodeposition.^20,21^ The pore sizes of these polymers can vary from a few nanometers to several tens of nanometers, depending primarily on the length and structure of the monomer used.^22–24^ Moreover, the electrical conductivity of these polymers has been shown to depend strongly on their redox state: while the reduced forms typically exhibit conductivities below 100 μS/cm, the oxidized forms display conductivities around 1 mS/cm.^20^

This review explores the conjugation properties of hydroxyl-functionalized azo dyes, provides an overview of their electropolymerization products as reported in the literature. It also offers suggestions for unreported products, examines their electrocatalytic sensor properties based on the calculations of extended Hückel charges–a semiempirical quantum chemistry method–and the Molecular Mechanics Force Field (MM2) method—a computational model used to describe the forces between atoms—for various analytes in real sample matrices, and evaluates the associated challenges and future perspectives (Figure 1). Beyond a mere literature review, this work explains the mechanisms underlying the electropolymerization of hydroxyl-functionalized azo-dye monomers and their electrocatalytic effects.

**Figure 1:**
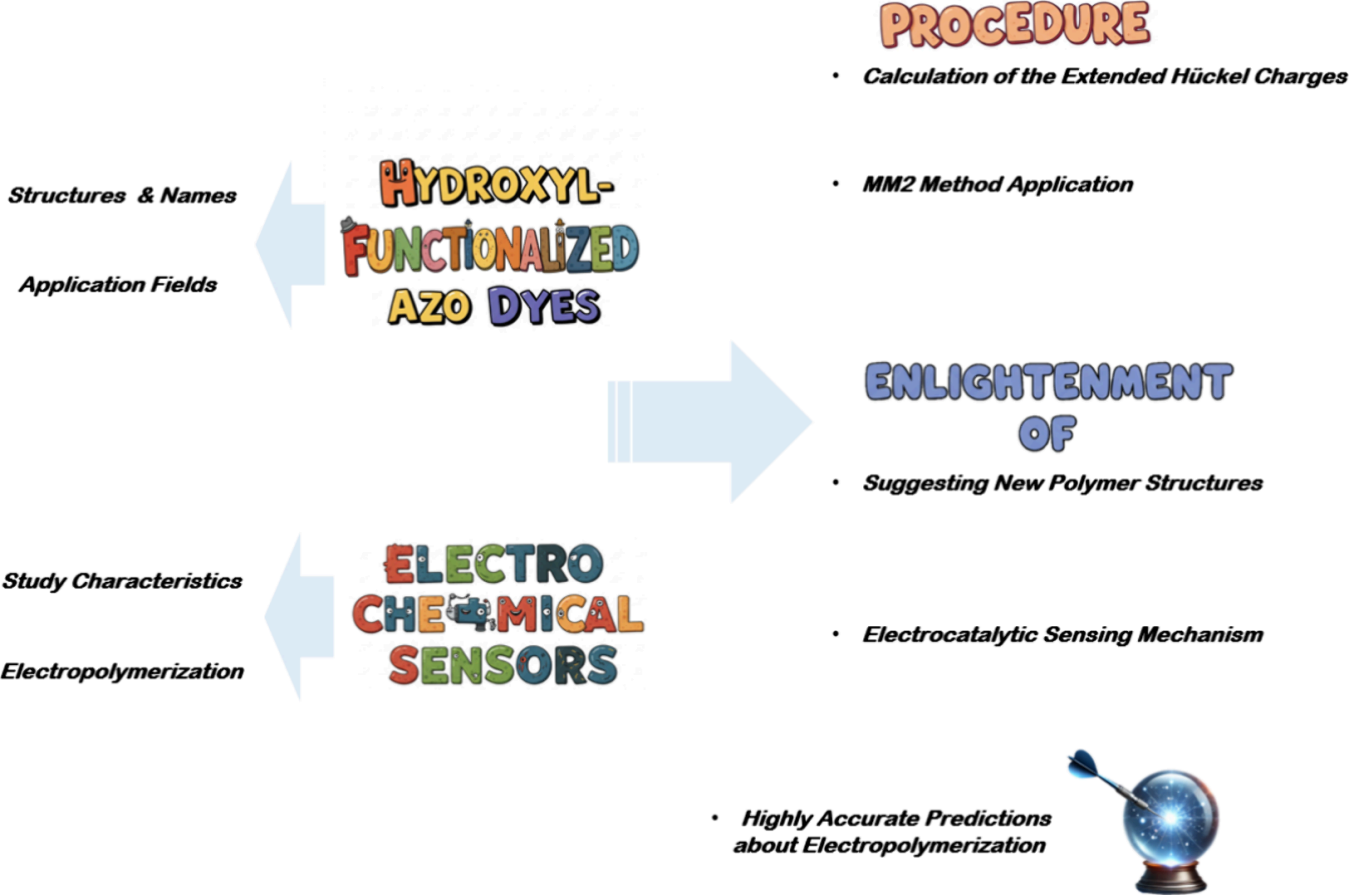
Contributions of this review study to the literature and its key findings (in summary: utilization of hydroxyl-functionalized azo dyes and their application areas, electropolymerization processes and characteristics, computational analyses explaining the electrocatalytic behavior of the polymers, elucidation of sensing mechanisms, and high-accuracy prediction of electropolymerization behavior without experimental procedures).

## 2 Hydroxyl-Functionalized Azo Dyes

Although numerous hydroxyl-functionalized azo dyes exist, this section focuses on monomers subjected to electropolymerization and utilized as electrochemical sensors. The structure, International Union of Pure and Applied Chemistry (IUPAC) name, common name, the abbreviation of the monomer name and main application fields of these monomers are provided in Table 1.

**Table 1:**
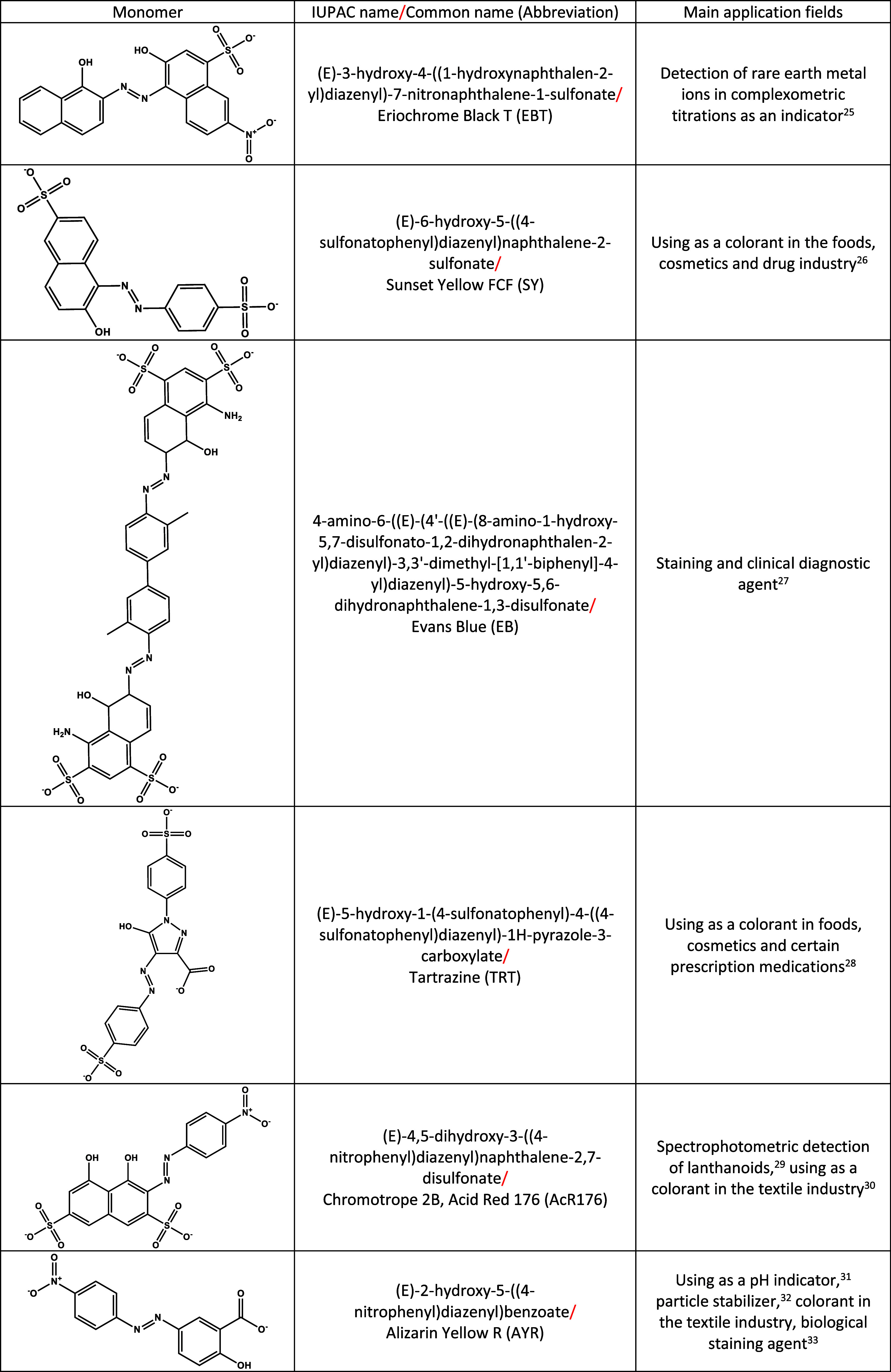

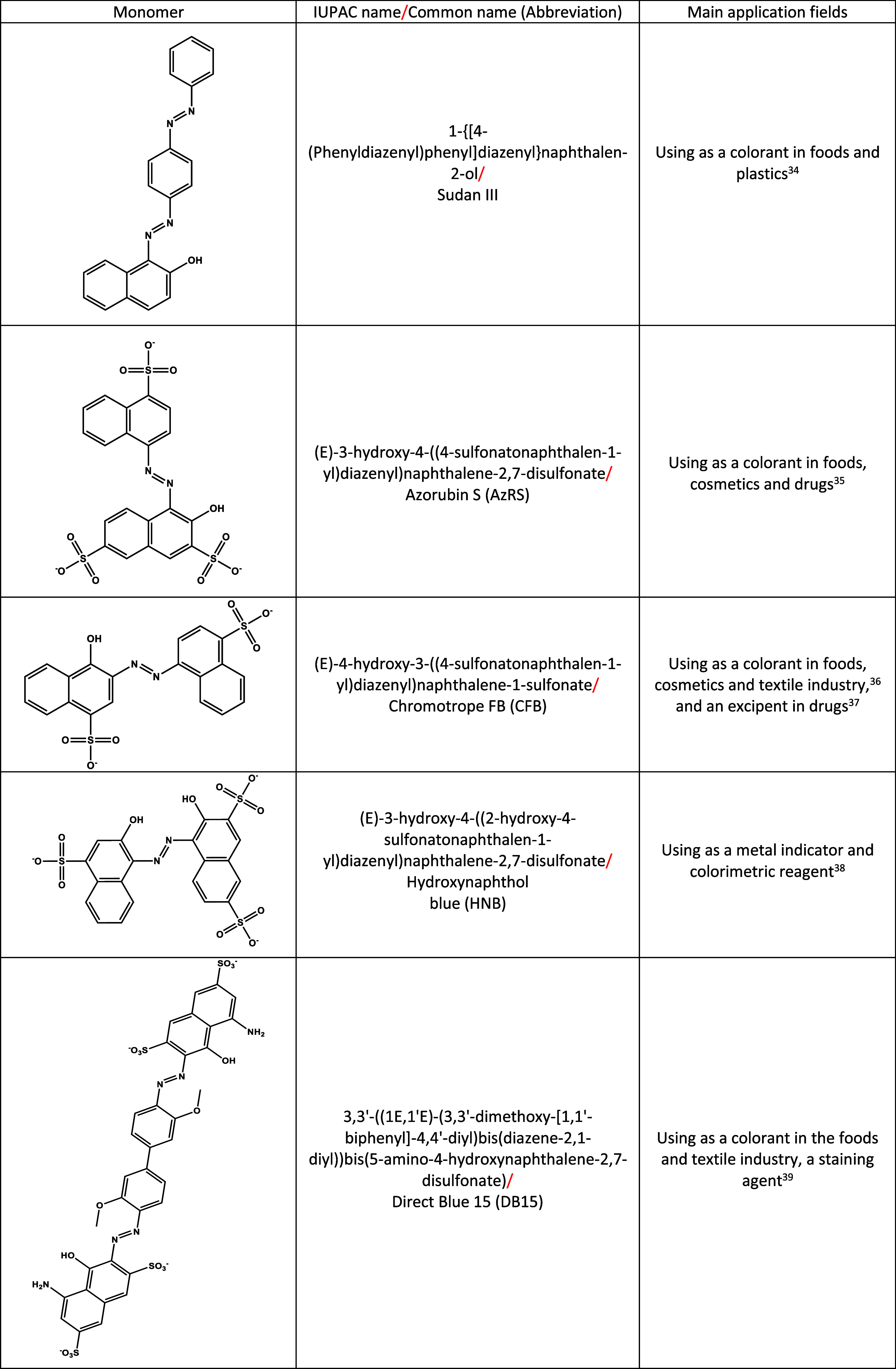

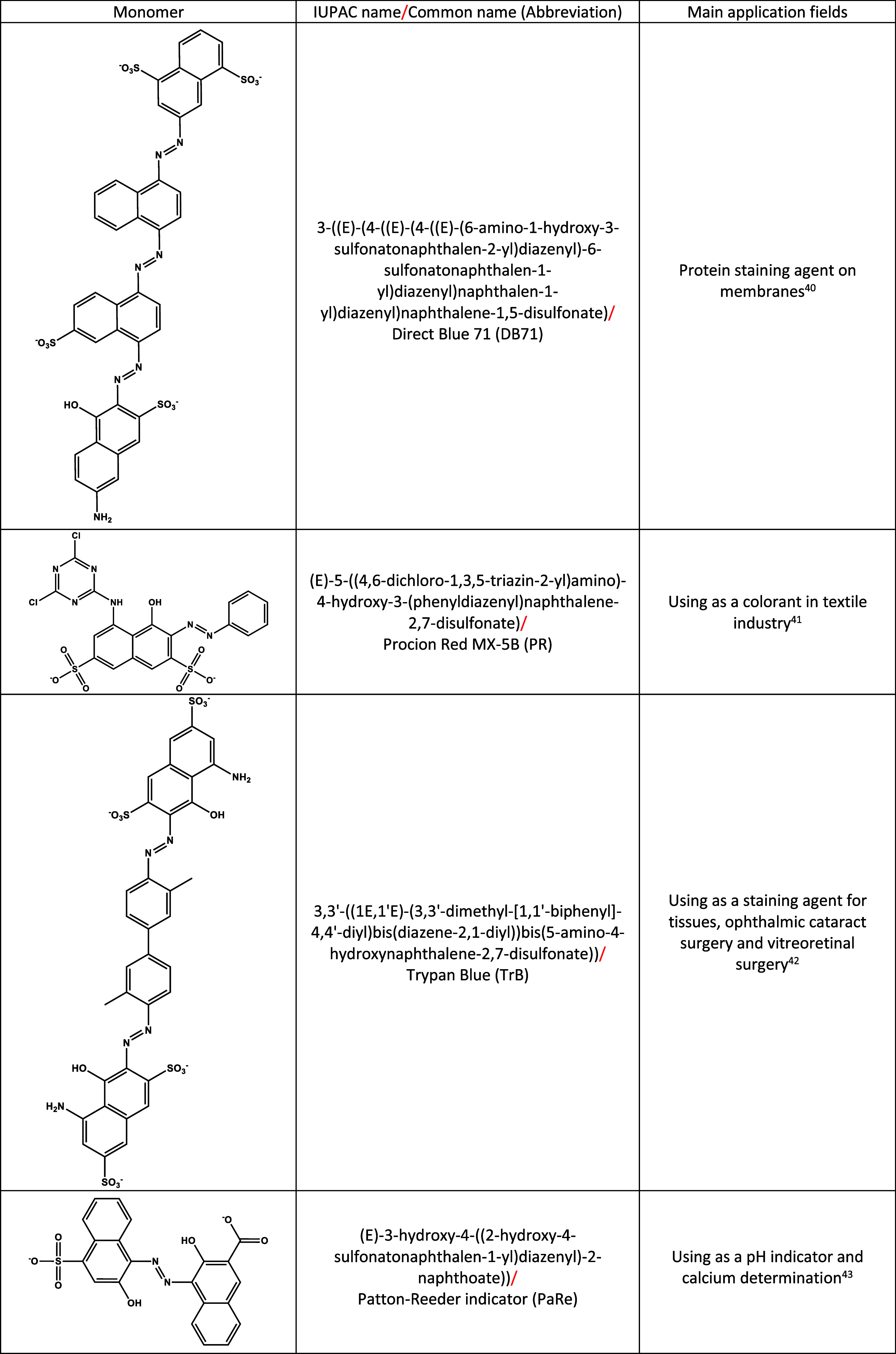

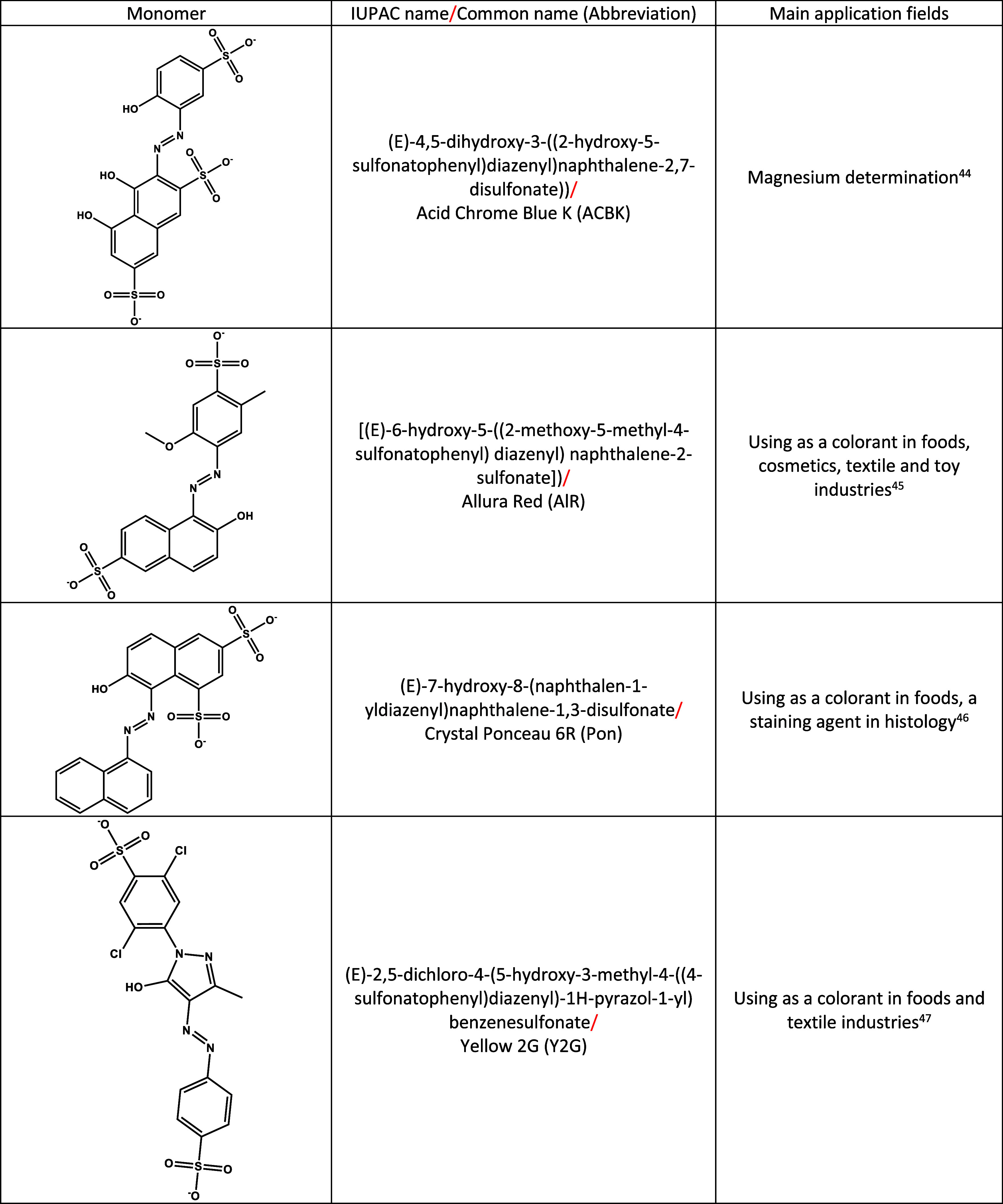
Monomer Structure, IUPAC and Common Names of the Monomers, and Main Application Fields of the Related Monomers^25,26,27,28,29,30,31,32,33,34,35,36,37,38,39,40,41,42,43,44,45,46,47^

## 3 Electrochemical Sensors Based on Hydroxyl-Functionalized Azo-Dye Polymers

It is important to distinguish between mere grafting of dye molecules onto the electrode surface and electropolymerization involving chain growth and electron-delocalized networks. In the reviewed studies, cyclic voltammetry (CV) technique was most commonly employed, typically yielding progressively increasing or decreasing redox peak currents that later stabilized, indicative of film formation on the electrode. The emergence of new redox peaks and potential shifts in several reports further corroborates the occurrence of polymer growth rather than simple dye immobilization.^9–11,13,15^ In one study utilizing chronoamperometry,^48^ the electropolymerization potential (1.1 V) corresponding to the monomer oxidation was applied until a steady-state current was achieved. These results provide additional evidence that the resulting coating originated from a chain-growth electropolymerization process rather than mere adsorption or grafting.^49,50,52,55,56,58^

In this section, a total of 47 hydroxyl-functionalized azo monomers from the literature, which have been utilized in electropolymerization-based sensors, have been reviewed. This analysis includes details on the monomer’s common name, supporting electrode material, sensor preparation and designation, proposed polymer structure, analyte, and sample application information, as presented in Table 2.

**Table 2:**
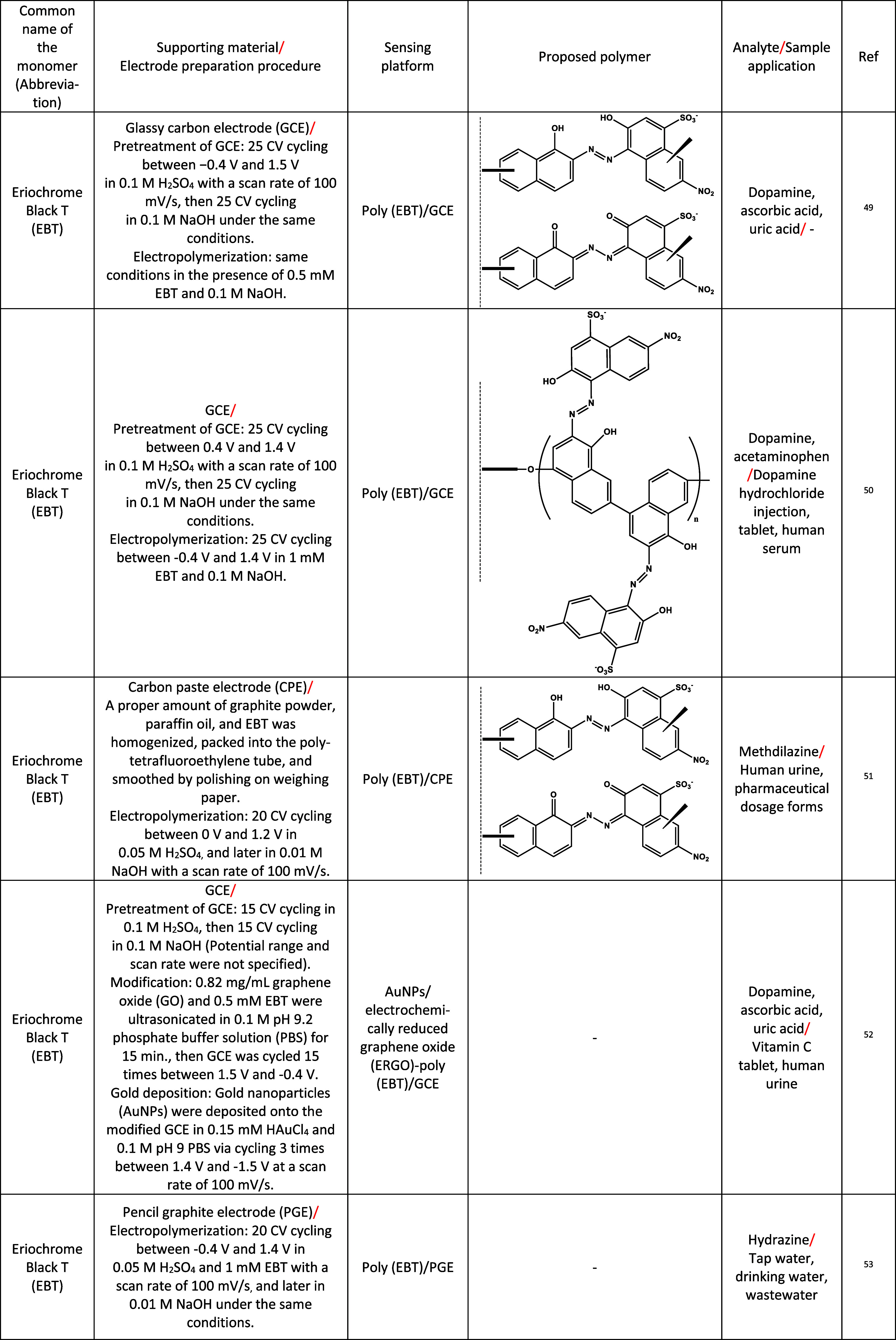

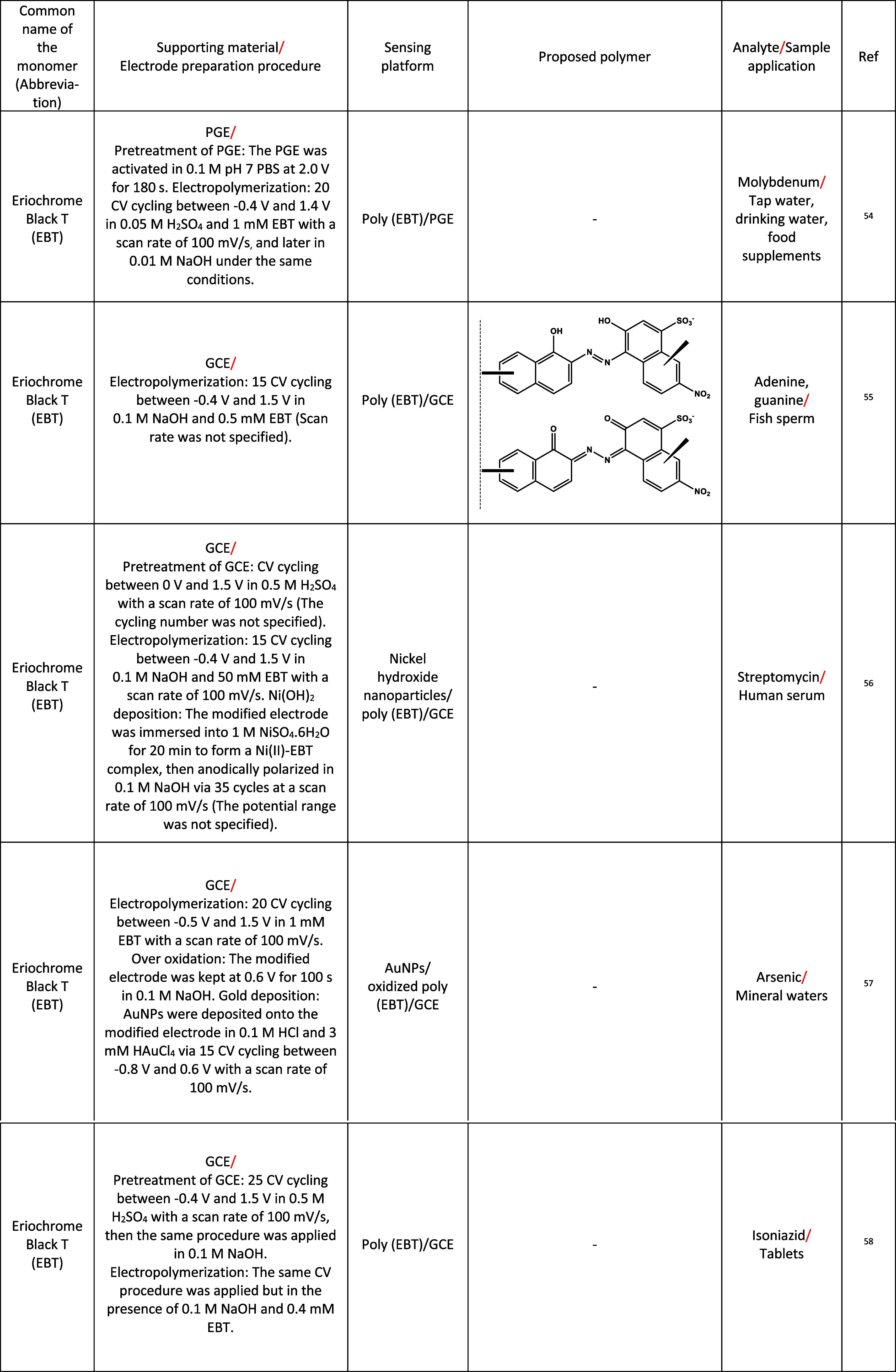

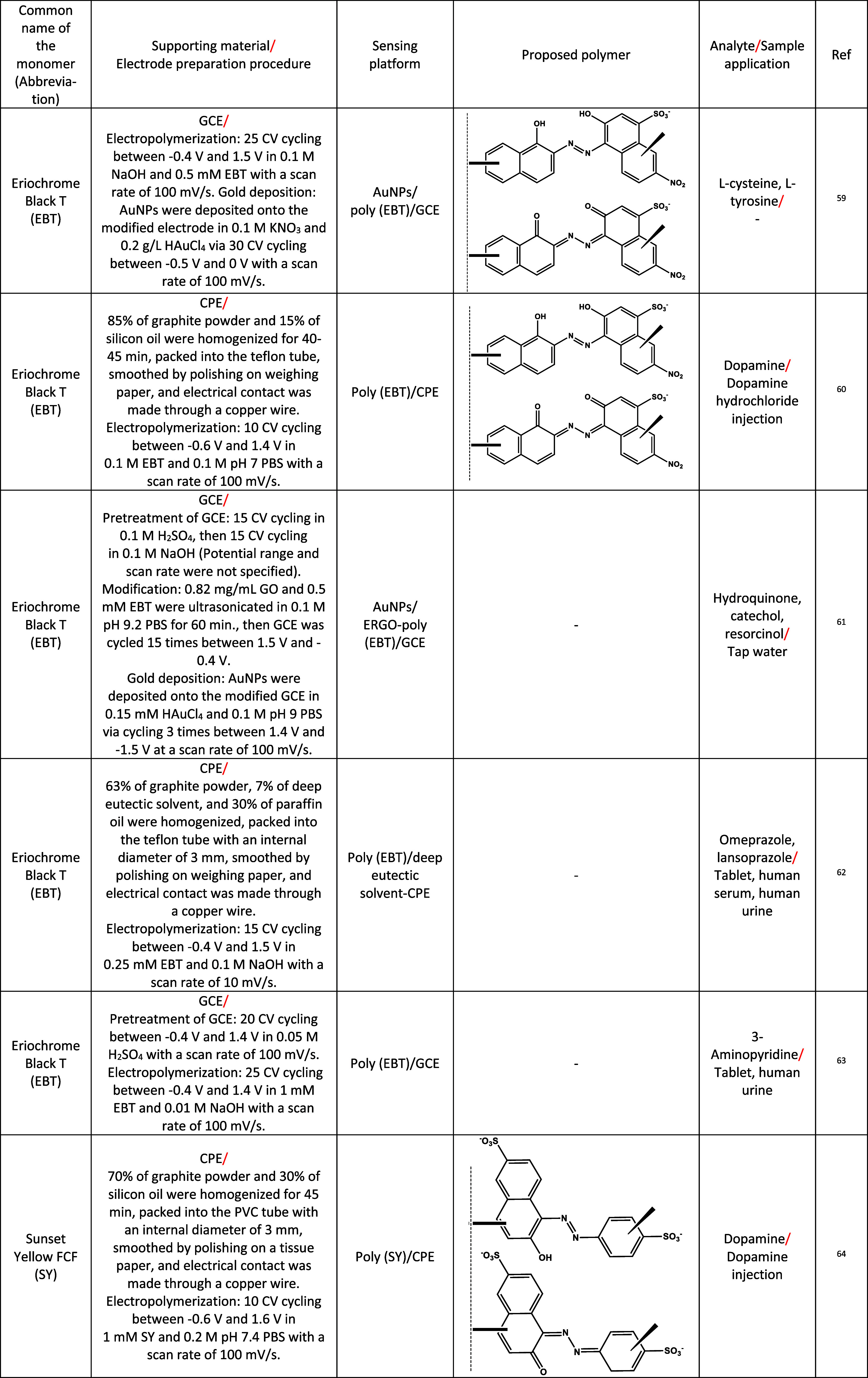

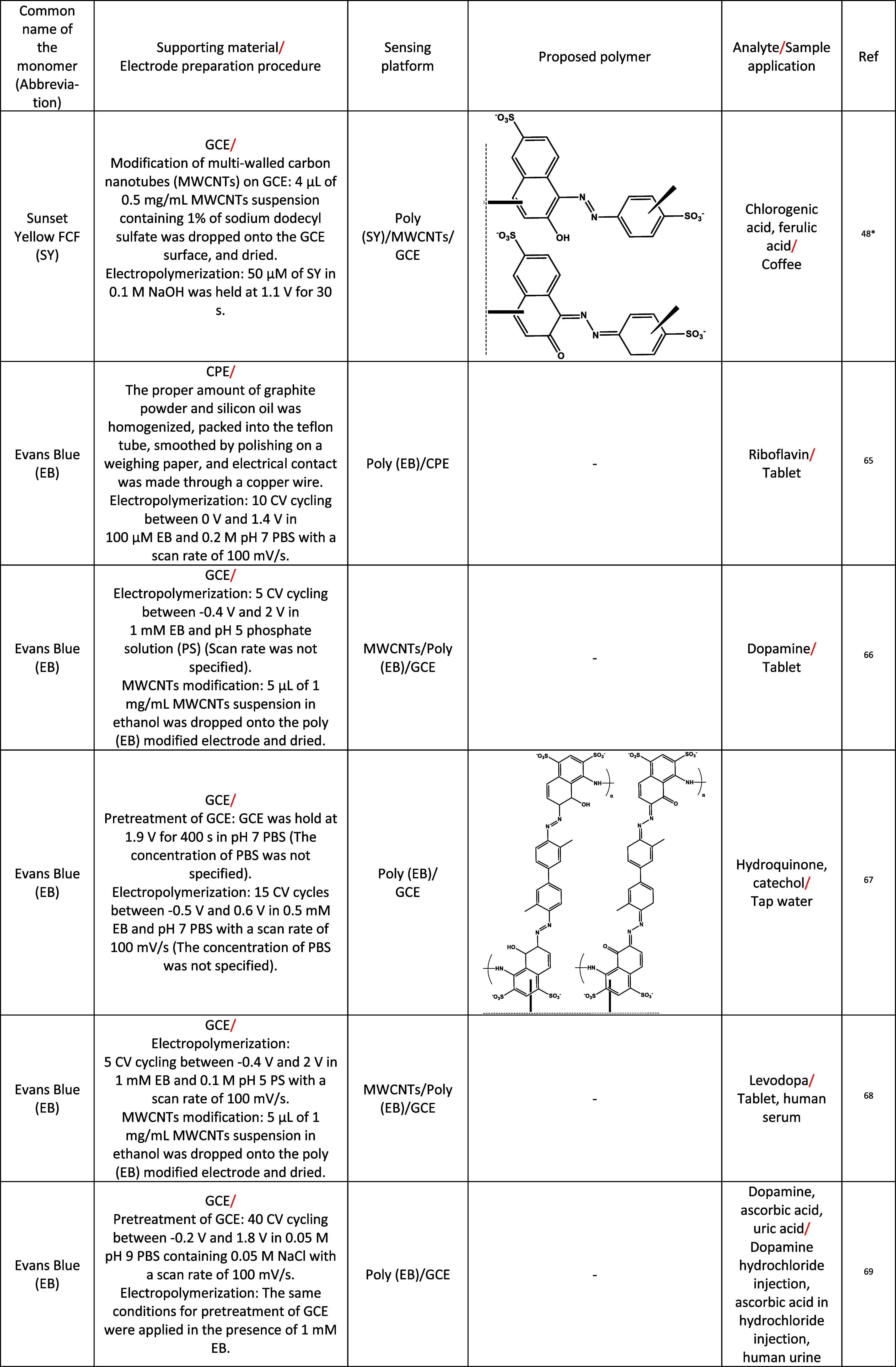

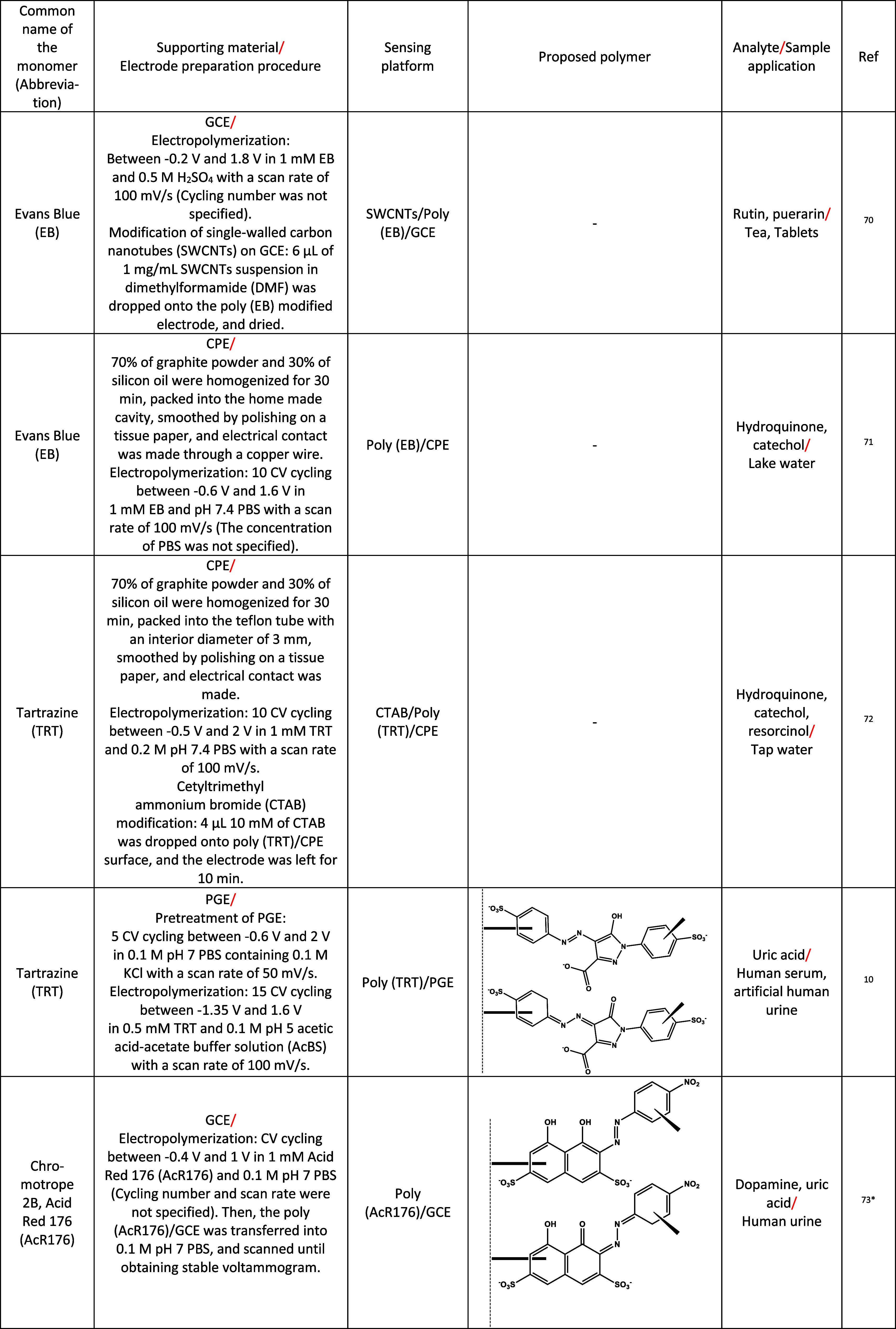

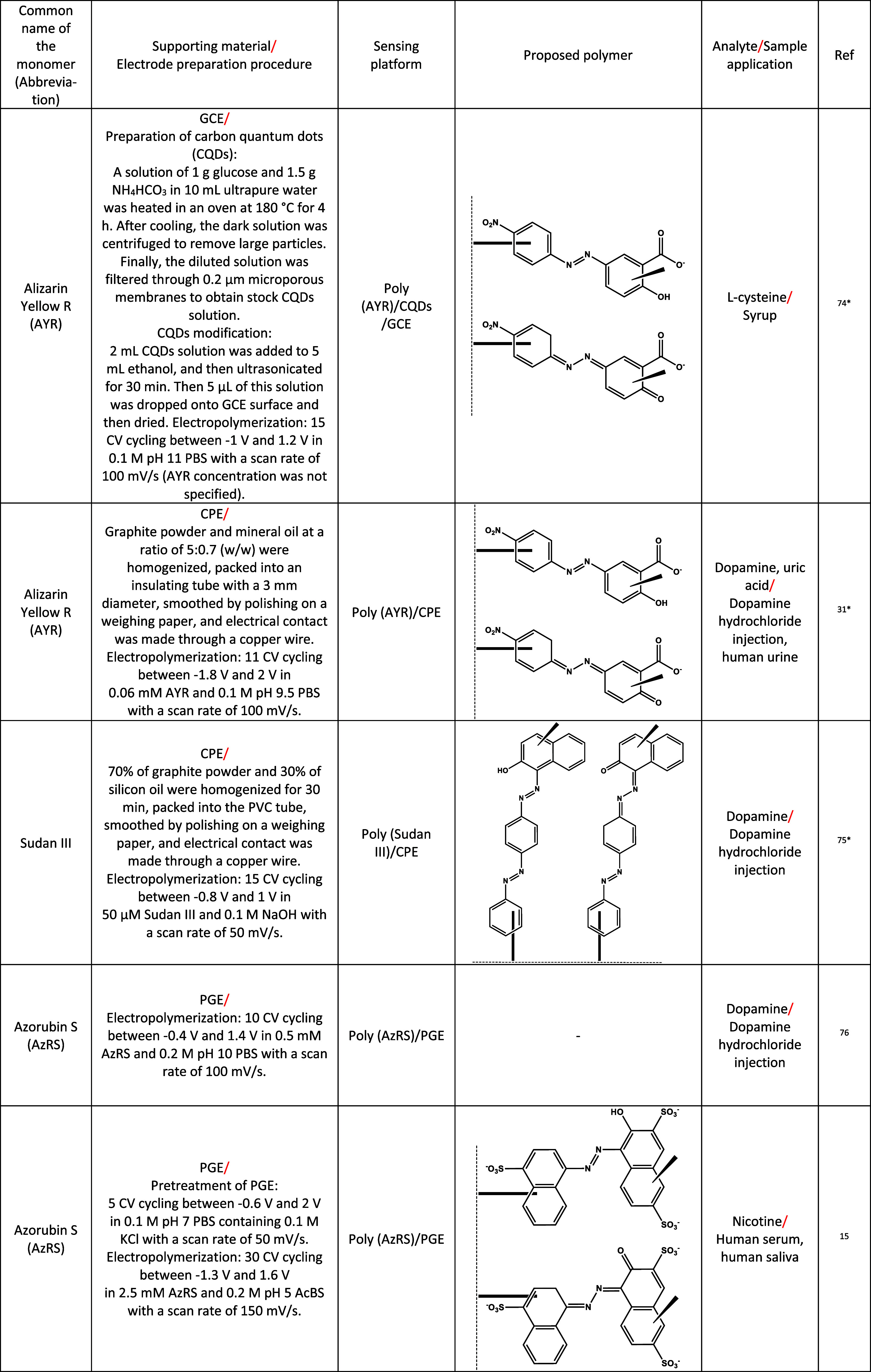

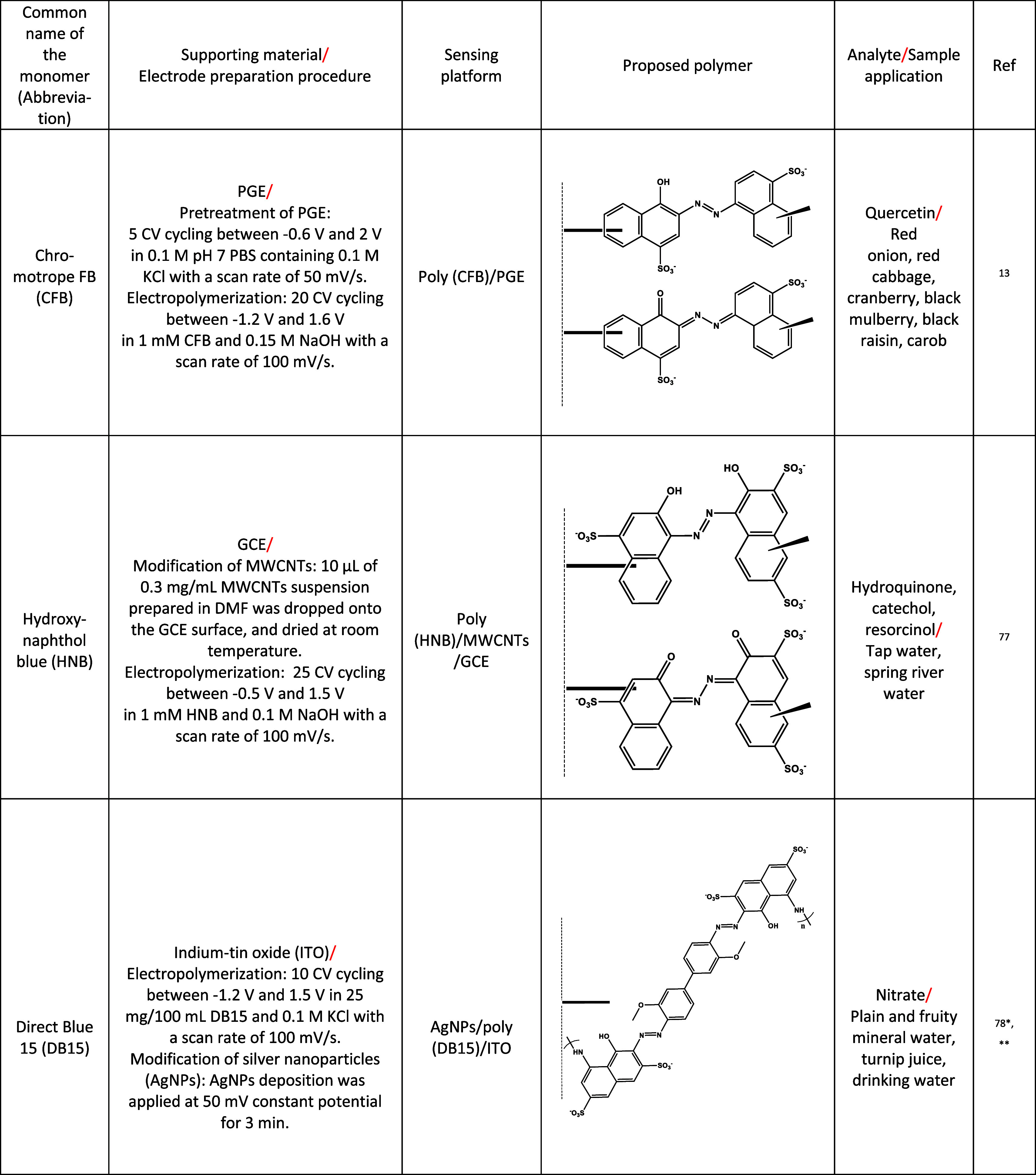

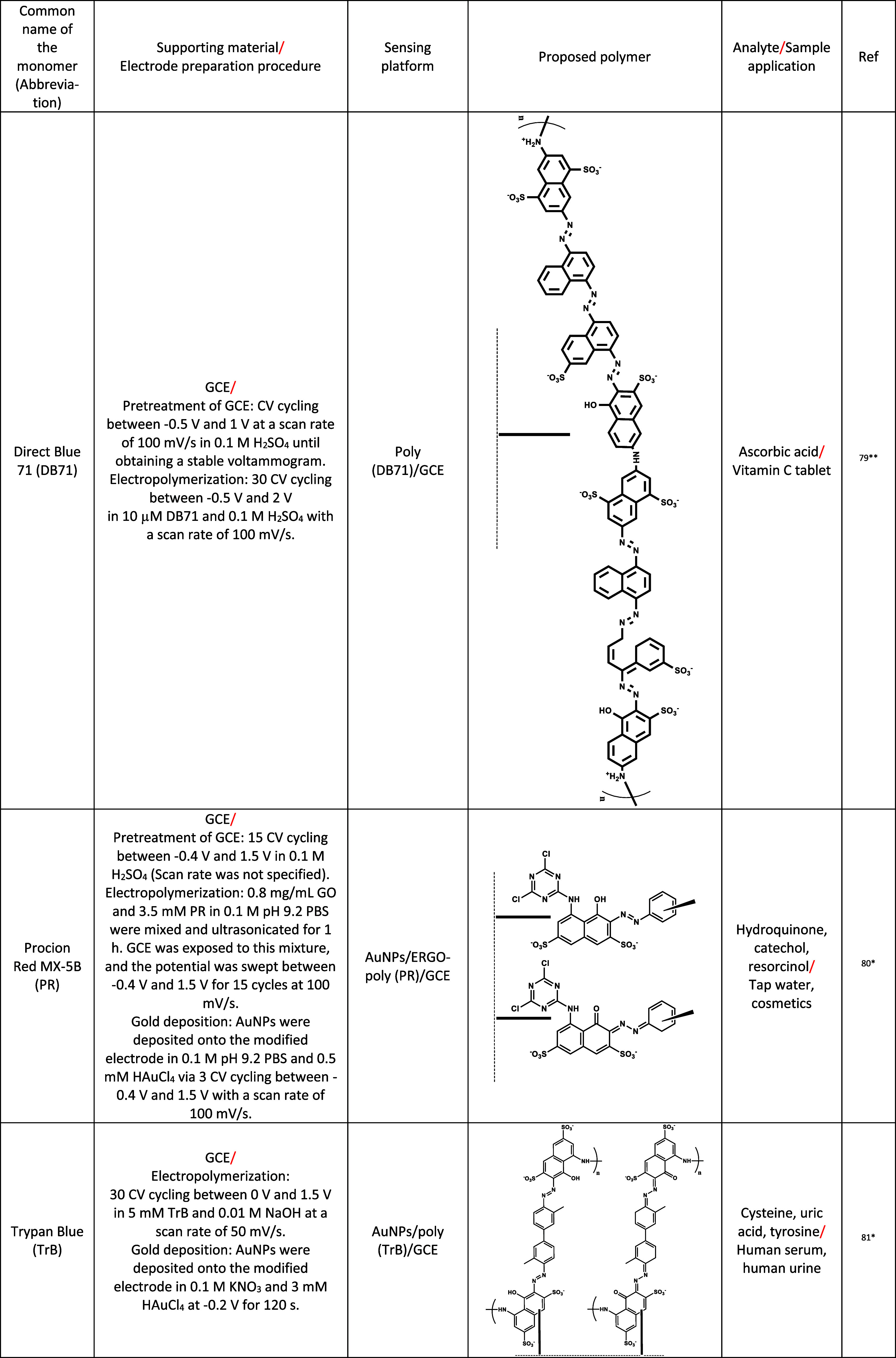

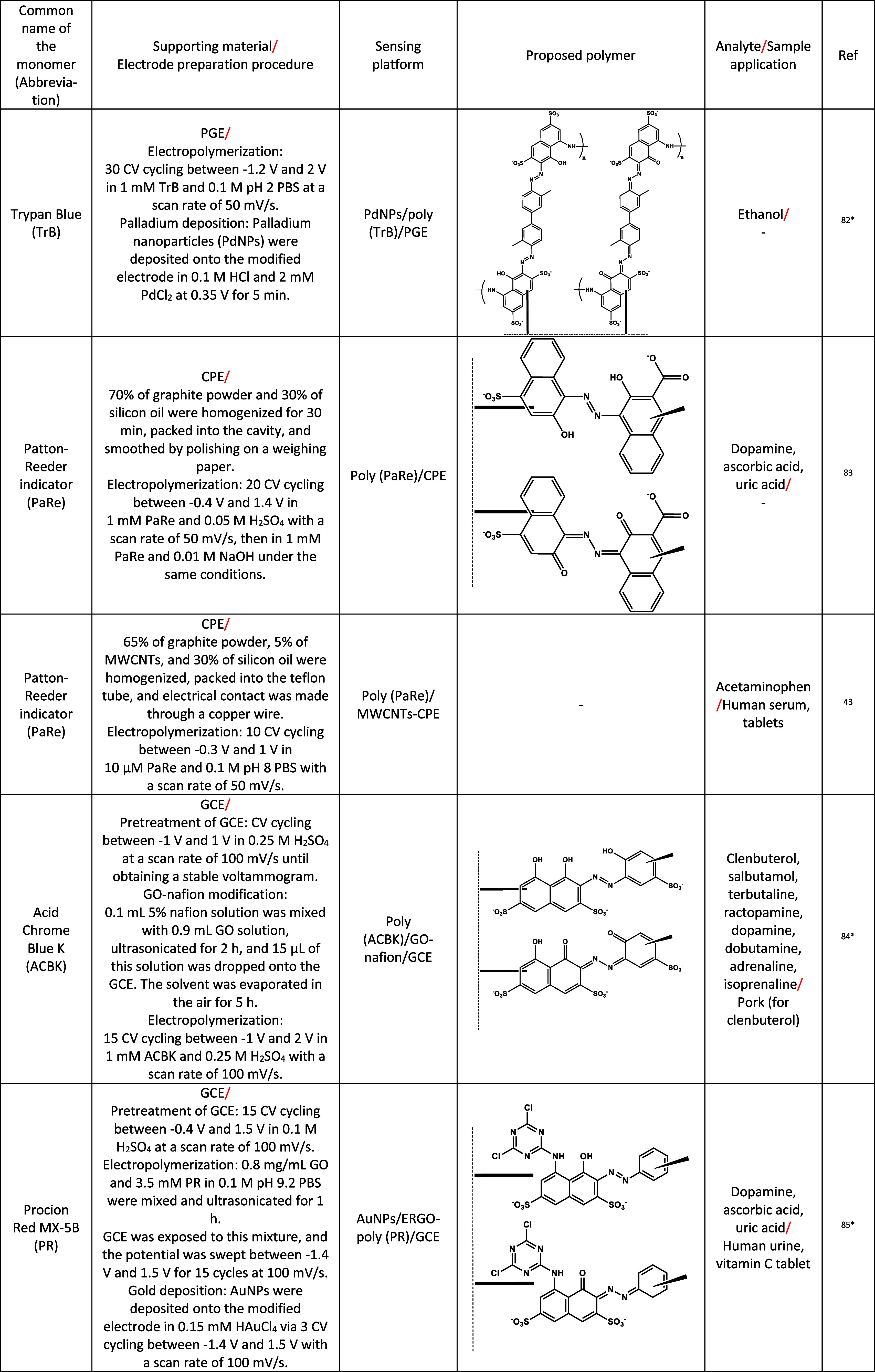

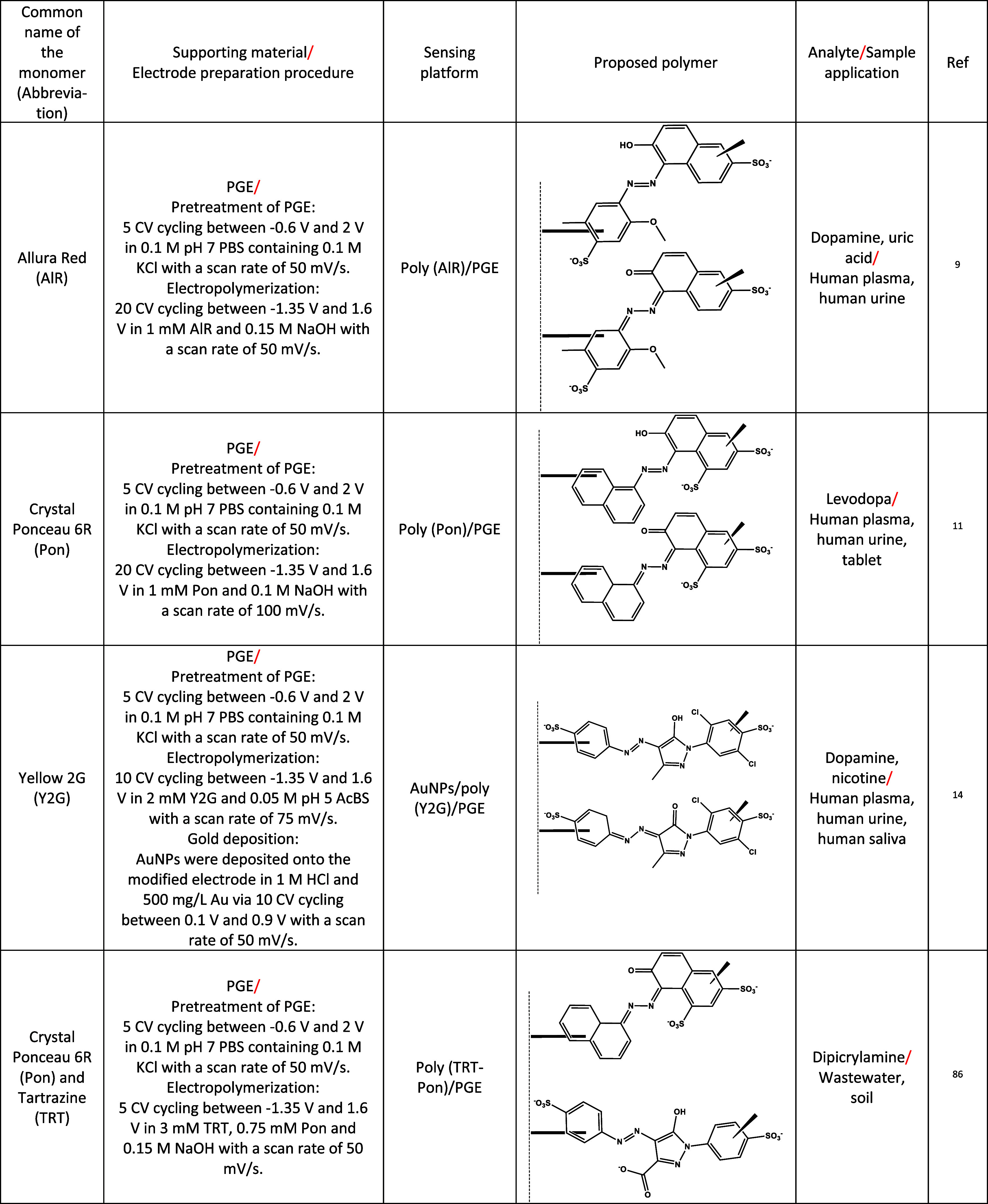
Electrochemical Sensing Applications of the Hydroxyl-Functionalized Azo-Dye Polymers (Used Monomer for Electropolymerization, Details of Electropolymerization Process, Proposed Polymer Structure on the Electrode Surface, the Analyzed Substance and Type of Real Sample)^a^ ^9,10,11,13,14,15,31,43,48,49,50,51,52,53,54,55,56,57,58,59,60,61,62,63,64,65,66,67,68,69,70,71,72,73,74,75,76,77,78,79,80,81,82,83,84,85,86^

a Asterisks (*) denote suggested polymer structures. Asterisks (**) denote only the reduced forms of the polymers, as the full structures are not fit within the table.

In the references section of Table 2, studies marked with (*) did not propose a polymer structure, so we suggested the polymer structures for these studies. While making these suggestions, it is proposed that when the structure contains only a hydroxyl functional group adjacent to the azo group, the azo group converts into imine structures neighboring the rings, while the hydroxyl group transforms into a ketone structure. The resulting polymer film consists of a mixture of structures containing azo-hydroxyl and imine-ketone groups, contributing to the overall enhanced electrocatalytic performance. On the other hand, electropolymerization is suggested to occur through amine groups if the monomer structure contains these groups attached to the aromatic ring(s) in addition to the hydroxyl group. However, it should be noted that the electropolymerization products may not correspond exactly to those presented in Table 2. The proposed polymeric structures represent the most plausible configurations, selected based on both our experimental findings and consistency with previously reported studies in the literature.^10,14,43,44,64,68,82^

Therefore, when the related mechanisms are examined in more detail, it can be stated that azo monomers containing hydroxyl functional groups—being generally aromatic and π-conjugated in nature—tend to adsorb onto carbon-based electrodes. This process is formed through π–π stacking interactions (between the aromatic monomer and the graphitic surface), hydrogen bonding (between the hydroxyl groups of the monomer and surface oxide functionalities), or electrostatic interactions.^87–89^ Subsequently, as illustrated in Figure 2, the monomer (**A**) undergoes deprotonation to form structure **B**, followed by a one-electron oxidation to yield a phenoxy radical (**C**). The **C** form can further delocalize its unpaired electron to generate structure **D**. Ultimately, attachment to the electrode surface may proceed via these **C** and **D** radical intermediates, while additional monomer molecules in the medium can also participate in chain propagation through radical coupling.^89^ In addition to covalent attachment, weaker physical interactions—such as π–π stacking, van der Waals forces, or hydrogen bonding—may also contribute to immobilization, albeit to a lesser extent. Because the exact binding site on the electrode surface cannot be definitively determined, the immobilization process is visually represented by thick black lines in Table 2.

**Figure 2:**
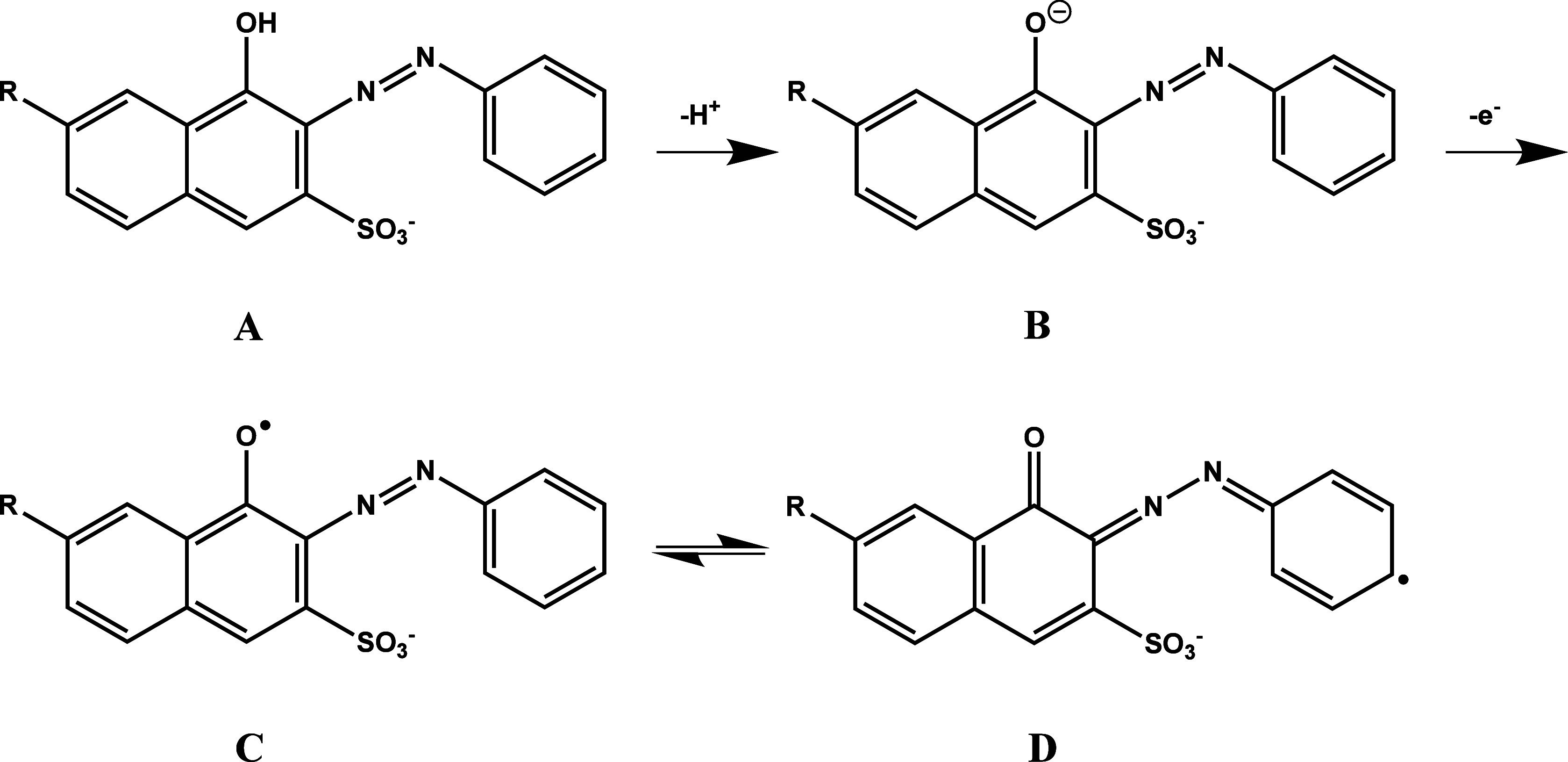
Proposed oxidation and radical formation process for hydroxyl-functionalized azo-dye monomer.

In cases where the monomer structure contains both amino and hydroxyl functional groups (Figure 3), the molecule (**I**) may lose one proton to form structure **II**, followed by a one-electron oxidation to generate radical intermediates **III** or **IV**.^67,79^ Under these conditions, in addition to the pathways described in Figure 2, polymer chain growth through the amino sites is also possible. However, according to previous studies,^67,79^ polymerization predominantly occurs through the amino termini of such monomers. As a result, the resulting film is typically durable, resistant to washing, and exhibits a semiconducting nature that facilitates efficient electron transfer.^10,11,86^

**Figure 3:**
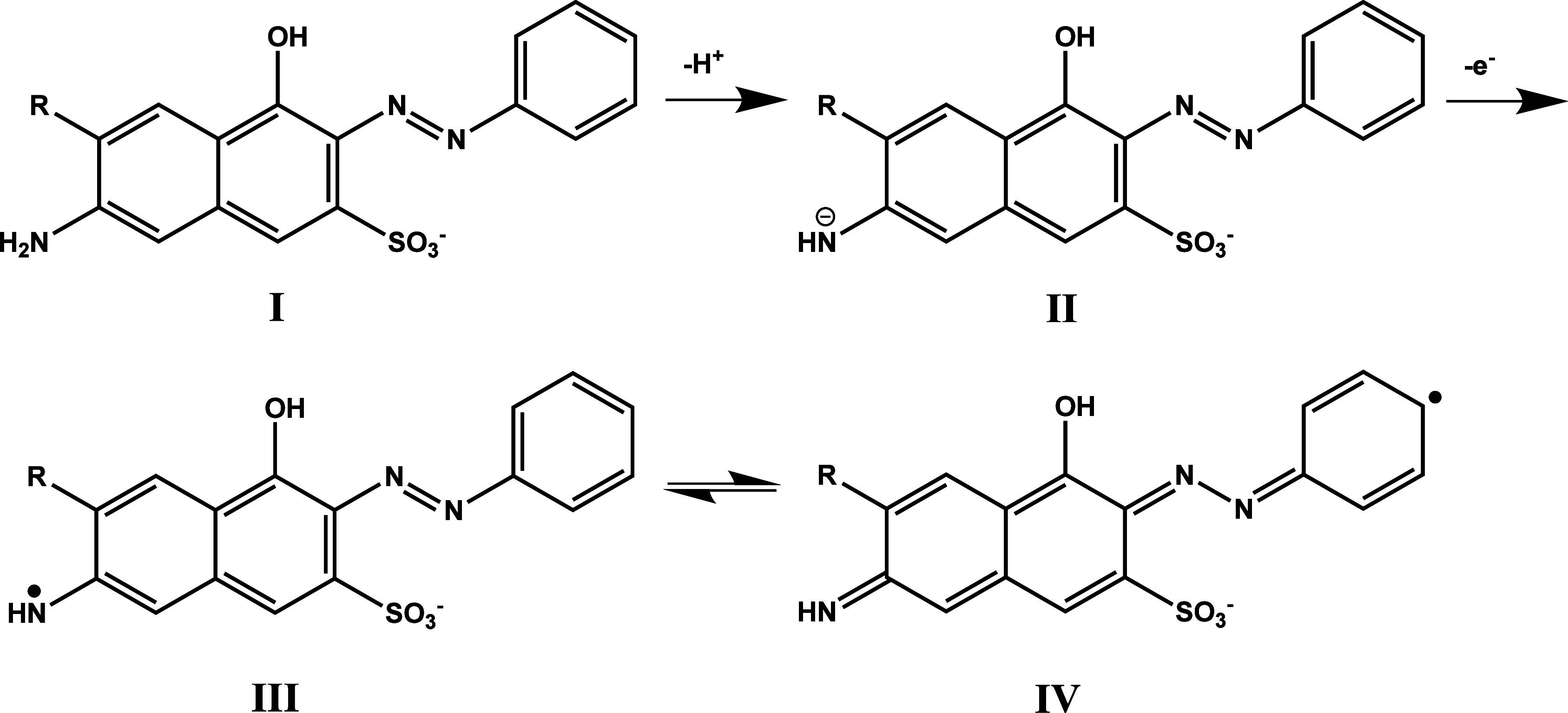
Proposed oxidation and radical formation process for amine- and hydroxyl-functionalized azo-dye monomer.

We also examined studies in the literature that report an increase in electrocatalytic current for polymer-film modified electrode (PFME) compared to bare electrode (BE), using data of peak potential, analyte type, and limit of detection (LOD) values (Table 3). These studies show that polymer structures significantly enhance sensitivity, and this improvement becomes even more pronounced when metal nanoparticles are incorporated into these structures.^14,57^

**Table 3:** Electrocatalytic Properties of Some Studies Based on Hydroxyl-Functionalized Azo-Dye Polymers (Comparison of Peak Potential and Current Responses of the Analyte Obtained at the Polymer Film–Modified Electrode Relative to the Bare Electrode, along with an Evaluation of the Sensitivity of the Analyzed Material)

PFME	BE	Increase in peak height (%)	Peak potential with PFME (mV)	Peak potential with BE (mV)	Analyte/LOD (nM)	ref
Poly(EBT)/CPE	CPE	332	675	685	Methdilazine/25.7	^51^
Poly(EBT)/PGE	PGE	96	550	550	Hydrazine/0.87	^53^
AuNPs/oxidized poly(EBT)/GCE	AuNPs/GCE	563	118	148	Arsenic(III)/ 77.5	^57^
Poly(EBT)/CPE	CPE	1700	230	–	Dopamine/800	^60^
Poly(EBT)/GCE	GCE	81	1005	1059	3-Aminopyridine/540	^63^
Poly(EB)/CPE	CPE	176	–485	–475	Riboflavin/210	^65^
Poly(TRT)/PGE	PGE	130	275	275	Uric acid/100	^10^
Poly(AYR)/CPE	CPE	500	170	400	Dopamine/600	^31^
Poly(AzRS)/PGE	PGE	410	700	700	Nicotine/89	^15^
Poly(CFB)/PGE	PGE	340	118	118	Quercetin/1.9	^13^
Poly(PaRe)/MWCNTs-CPE	CPE	101	462	455	Acetaminophen/530	^43^
Poly(AlR)/PGE	PGE	344	190	185	Dopamine, uric acid/10.5, 30.1	^9^
		792	320	240		
Poly(Pon)/PGE	PGE	403	180	290	Levodopa/20	^11^
AuNPs/poly(Y2G)/PGE	PGE	204	140	220	Dopamine, nicotine/58.8, 7.2	^14^
		526	960	840		
Poly(TRT-Pon)/PGE	PGE	346	–525	–507	Dipicrylamine/57	^86^

In the context of Table 3, considering three main electrochemical criteria—the lower LOD, the higher increase in peak current relative to the bare electrode, and the shift of the oxidation/reduction potential toward lower/higher values—the poly ponceau-film modified electrode (poly­(Pon)/PGE) exhibits the most favorable performance.^11^ Structural analysis of the azo-dye monomers reveals that yellow 2g (Y2G) and tartrazine (TRT) have an electron withdrawing pyrazole ring adjacent to the azo linkage, which typically hinders electron delocalization and catalytic activity. In chromotrope fb (CFB) and eriochrome black t (EBT), the presence of strong deactivating sulfonate groups reduces the electron density on the aromatic rings. Moreover, EBT also contains a nitro group, further diminishing its reactivity. Similarly, azorubin s (AzRS) and Patton–Reeder (PaRe) dyes possess sulfonate groups on the hydroxyl-bearing rings, leading to deactivation of the benzene ring. Among the remaining dyes—evans blue (EB), allura red (AlR), and ponceau (Pon)—although these molecules also contain deactivating sulfonate groups, EB is the most sterically hindered, with bulky substituents that limit the accessibility of its active sites. When comparing AlR and Pon, the latter exhibits a more symmetric and planar molecular structure, allowing better conjugation and more exposed polymerization-active sites. In both molecules, the sulfonate groups are not located on the benzene ring bearing the hydroxyl group. However, the presence of an additional benzene ring in Pon enables a higher degree of electron delocalization. These structural features likely account for the superior electrocatalytic performance of the poly­(Pon) film.

## 4 Electrocatalytic Sensing Mechanism of Hydroxyl-Functionalized Azo-Dye Polymers

After the electropolymerization of monomers containing hydroxyl and azo functional groups, the resulting polymer structure significantly enhances the reduction or oxidation peak currents of the target analyte. This phenomenon is believed to stem from the ability of the azo and hydroxyl functional groups within the polymer structure to facilitate electron and/or proton transfer efficiently. A review of the studies in Table 2 indicates that proton transfer is generally as crucial as electron transfer for their detection. Consequently, the electrocatalytic effect becomes even more pronounced in electrode reactions where proton transfer is also required. A possible mechanism for this process is illustrated in Figure 4, which depicts dopamine oxidation on a poly­(EBT) film (containing azo and hydroxyl groups) and a poly­(TrB) film (containing azo, hydroxyl, and amine groups). In this case, the two electrons and two protons released during dopamine oxidation are immediately utilized by the poly­(EBT) and poly­(TrB) films, thereby triggering the electrocatalytic effect. In both cases, electron and proton transfer must occur in the regions where the azo and hydroxyl functional groups are present (red and green regions).

**Figure 4:**
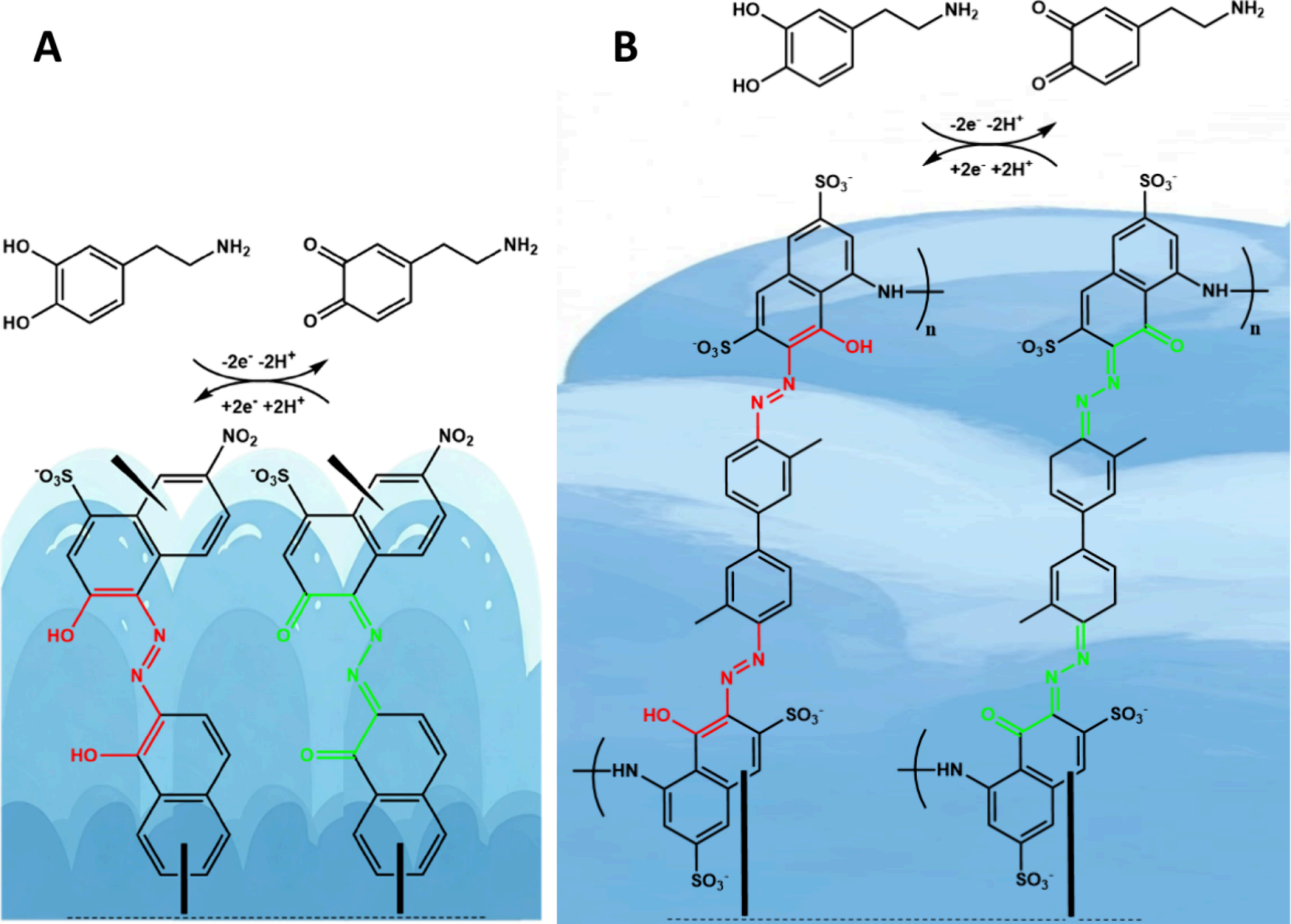
(A) Possible polymer film of EBT and (B) TrB, and oxidation mechanism of dopamine along with reduction process of polymer films (electron and proton transfer between polymer fragments and the consequent catalytic enhancement of the redox process).

Apart from dopamine, the electrochemical oxidation or reduction mechanisms of all other analytes reported in the literature using hydroxyl-functionalized azo-dye polymers are presented in Figure S1. In addition to these redox mechanisms, the overall reversibility, irreversibility, and quasi-reversibility of the reactions were also evaluated. We proposed novel electrochemical pathways for the detection of analytes such as lansoprazole, 3-aminopyridine, ferulic acid, clenbuterol, dobutamine, and streptomycin whose determination mechanisms were not previously described in the literature (Figure S1).

Accordingly, molecules such as ascorbic acid,^90^ acetaminophen,^91^ hydroquinone, catechol,^61^ chlorogenic acid,^48^ rutin,^92^ dobutamine, adrenaline,^93^ and isoprenaline^94^ are presumed to undergo oxidation through their hydroxyl groups, generally exhibiting reversible electrode reactions. In contrast, although levodopa^11^ and quercetin^13^ share structural similarities and are oxidized via hydroxyl groups, their electrode processes typically proceed in a quasi-reversible manner. It is most likely due to the limited solubility of their oxidation products in the solvent medium. Similarly, molecules such as l-tyrosine,^59^ puerarin,^95^ ferulic acid, terbutaline,^96^ and ractopamine^97^ also undergo hydroxyl group oxidation but follow irreversible electrode processes. Compounds such as resorcinol^61^ and salbutamol,^98^ which can generate radicals during hydroxyl group oxidation and subsequently polymerize, were also investigated for analytical purposes. An another representative example of radical-based oxidation is methdilazine,^99^ which undergoes irreversible oxidation through the sulfur atom of its phenothiazine ring to generate a radical species (Figure S1).

Beyond these cases, compounds such as omeprazole,^100^ lansoprazole, and 3-aminopyridine undergo hydroxylation in aqueous media, followed by irreversible oxidation to yield ketone functionalities. Adenine and guanine^101^ display redox processes similar to prazole derivatives in their initial steps, involving water addition and subsequent irreversible oxidation. However, in their final step, hydrogens bound to the nitrogen atoms of the purine ring are eliminated, leading to a reversible redox reaction (Figure S1).

Apart from the molecules listed above, studies on other compounds reveal distinct redox mechanisms. For instance, isoniazid^102^ oxidation proceeds via the loss of two protons and electrons from the carbohydrazide group, forming a double bond between nitrogen atoms, followed by chemical decomposition to release nitrogen gas and produce 4-pyridinecarboxylic acid. In l-cysteine,^59^ the presence of thiol groups results in proton loss from these groups during oxidation, forming cystine. Nicotine^14,103^ oxidation has been explained by several proposed pathways, the most plausible involving the loss of equal numbers of protons and electrons, incorporation of a hydroxyl group, and simultaneous release of methanol. For clenbuterol, the oxidation process likely involves the loss of equal numbers of protons and electrons, with a water molecule binding at the amino terminus, yielding a hydroxylamine derivative. Streptomycin, being protonated at its guanidine groups at physiological pH, is considered to undergo a reversible redox process through the loss of these protons. Riboflavin,^104^ in contrast, is reduced via the gain of two protons and two electrons at its benzo­[g]­pteridine moiety. All of the redox reactions described in this section are essentially irreversible except for streptomycin (Figure S1).

Furthermore, hydroxyl-functionalized azo polymer film electrodes can be employed for the determination of transition metals or their different forms by facilitating the oxidation of small organic molecules capable of forming complexes with these metals. For example, molybdate can complex with tiron, followed by oxidation through the hydroxyl terminals of tiron, leading to complex dissociation.^54^ Hydrazine undergoes irreversible oxidation by donating four protons and four electrons to release nitrogen gas.^53^ Nitrate is irreversibly reduced by two electrons to nitrite, and upon further potential scanning, can accept an additional four electrons to form hydroxylamine.^105^ Ethanol oxidation, depending on the alkalinity of the medium, yields acetaldehyde, acetic acid, or carbon dioxide.^106^ In addition, due to the electron-mediating capability of these polymer films, anodic stripping voltammetry can be applied for the determination of metal cations (Figure S1).^57^

Taken together, the evaluation of all mechanisms provides clearer evidence that the protons and electrons generated or consumed by the polymer films are responsible for the observed electrocatalytic effect. It should also be noted that an increase in the effective surface area does not occur for each polymer film.

## 5 The Rationale Behind the Electrocatalytic Effect: Comprehensive Calculations

This section aims to illuminate the underlying truth behind the electrocatalytic effect of platforms created by the electropolymerization of hydroxyl-functionalized azo dyes. To achieve this, the calculation of extended Hückel charges and the MM2 method was used comparatively, both in the presence and absence of hydroxyl groups in the relevant azo dyes, utilizing ChemBio3D Ultra 14.0 software. In the extended Hückel method unlike the conventional Hückel approach, both σ (sigma) and π (pi) orbitals are taken into account. In this study, the standard extended Hückel method was employed, with the Wolfsberg–Helmholz constant set to 1.75. For the MM2 calculations, the following parameters were applied: cubic stretch constant = −2.000, quartic stretch constant = 2.333, X–B, C, N, O–Y stretch–bend interaction force constant = 0.120, X–B, C, N, O–H stretch–bend interaction force constant = 0.090, X–Al, S–Y stretch–bend force constant = 0.250, sextic bending constant = 7 × 10^–8^, and dielectric constant for charges and dipoles = 1.500. The cutoff distances were defined as follows: 35.000 Å for charge–charge interactions, 25.000 Å for charge–dipole interactions, 18.000 Å for dipole–dipole interactions, and 10.000 Å for van der Waals interactions. Each of these parameters was assigned a quality level of 4, indicating that the corresponding values are experimentally determined and validated.

The extended Hückel charges of the relevant molecules were calculated and are presented in Table 4. In this table, N1, N2, N3, and N4 represent the azo nitrogen, (N)-C refers to the carbon adjacent to the azo group and the hydroxyl-bearing carbon, while (O)-C denotes the carbon atom bonded to the hydroxyl group. In the notation used, the direct use of the monomer abbreviation refers to the original monomer containing the hydroxyl group, whereas the subscript ‘na’ denotes the corresponding form of that monomer lacking the hydroxyl group. Examining the (O)-C extended Hückel charges, it is observed that higher values are obtained when the hydroxyl group is present. This is due to oxygen withdrawing electron density from the bonded carbon, making that carbon more positively charged. On the other hand, when analyzing (N)-C extended Hückel charges, generally lower values are observed in hydroxyl-containing monomers (Only in the monomers of AzRS and EBT was a minimal decrease observed, while SY showed no change). This can be attributed to the conjugation between the oxygen’s lone pairs, the benzene ring, and the azo group’s π-bond, which increases electron localization in this region. As a result, the carbon bonded to the azo group carries a lower charge in hydroxyl containing monomers (the numbering was assigned according to IUPAC conventions for molecules with two hydroxyl groups, where the first azo group in the structure was considered the reference as shown in the first rows of Table 4).

**Table 4:** Extended Hückel Charge Results for Azo-Dye Monomers with (Denoted as Monomer) and without Hydroxyl Groups (Denoted as Monomer_na_) (Demonstration of the Active Role of Hydroxyl Groups in the Electrocatalytic Effect through the Analysis of Extended Hückel Charges of Azo Groups and Their Adjacent Carbon and Hydroxyl Atoms)

Monomer	N1	N2	N3	N4	(N)-C	(O)-C
ACBK	0.848	0.527			0.116	0.389
					0.233	0.236
ACBK_na_	0.816	0.863			0.234	0.074
					0.194	0.005
ACR176	0.833	0.730			0.091	0.310
ACR176_na_	0.018	0.016			0.178	–0.007
AlR	0.727	0.804			0.100	0.250
AlR_na_	0.761	0.821			0.177	–0.035
AYR	0.850	0.763			0.137	0.304
AYR_na_	0.831	0.816			0.178	0.010
AzRS	0.772	0.962			0.327	0.431
AzRS_na_	0.740	0.907			0.303	0.049
CFB	0.743	0.785			0.092	0.278
CFB_na_	0.738	0.833			0.170	–0.033
DB15	0.729	0.777	0.684	0.791	0.077	0.256
					0.069	0.292
DB15_na_	2.633	2.633	–0.134	0.037	0.141	–0.033
					0.034	–0.073
DB71	0.689	0.672			0.066	0.229
DB71_na_	0.729	0.636			0.130	–0.115
EBT	0.767	0.091			0.021	0.271
					–0.002	0.265
EBT_na_	0.016	0.786			0.018	0.003
					–0.032	0.175
EB	0.703	0.932	0.545	0.836	0.132	0.209
					0.136	0.198
EB_na_	0.664	0.943	0.671	0.939	0.151	–0.063
					0.038	0.038
HNB	0.916	0.222			0.260	0.415
					0.077	0.056
HNB_na_	0.177	0.081			0.303	0.049
					0.060	–0.027
PaRe	0.553	0.658			0.033	0.196
					0.037	0.173
PaRe_na_	0.543	0.618			0.085	–0.164
					0.085	–0.166
Pon	0.801	0.721			0.089	0.281
Pon_na_	0.011	0.014			0.170	–0.015
PR	0.798	0.700			0.069	0.305
PR_na_	0.817	0.735			0.125	–0.016
Sudan III	0.606	–0.098			–0.047	0.088
Sudan III_na_	–0.103	0.015			–0.021	–0.241
SY	–0.077	–0.191			0.016	0.060
SY_na_	–0.031	0.021			0.016	0.057
TRT	0.824	0.699			0.140	0.372
TRT_na_	0.829	0.753			0.190	0.046
TrB	0.783	0.726	0.811	0.653	0.071	0.277
					0.090	0.291
TrB_na_	0.765	0.793	0.832	0.735	0.140	–0.026
					–0.013	0.195
Y2G	0.390	0.502			–0.046	0.005
Y2G_na_	0.380	0.520			0.005	–0.328

The MM2 method represents molecules’ total potential energy, or steric energy. Calculations were performed for all monomers in the literature, both in the presence of hydroxyl groups (denoted as Monomer) and in their absence (denoted as Monomer_na_), with the results provided in Table 5. The parameters in this table are defined as follows:

- “Stretch”: Energy related to deviations of bond lengths from their optimal values.
- “Bend”: Energy associated with deviations of bond angles from their optimal values.
- “Stretch–bend”: Energy required to stretch two bonds within a bond angle when the angle is significantly compressed.
- “Torsion”: Energy related to deviations of torsional angles in the molecule from their ideal values.
- “Non-1,4 VDW (van der Waals)”: Energy for interactions between atom pairs separated by more than three atoms, mediated by intermolecular magnetic or electronic fields.
- “1,4 VDW”: Energy for interactions between atoms separated by two atoms, influenced by intermolecular magnetic or electronic fields.
- “Dipole/dipole”: Steric energy related to interactions between bond dipoles.
- “Charge/charge”: Steric energy from interactions between charges at two points.
- “Charge/dipole”: Steric energy based on the combined interaction of partial charges with bond dipoles and charged groups.

**Table 5:** The Results of the MM2 Method for Azo-Dye Monomers with (Denoted as Monomer) and without Hydroxyl Groups (Denoted as Monomer_na_) (Demonstration of the Active Role of Hydroxyl Groups in the Electrocatalytic Effect through MM2-Based Analysis of the Various Energy Forms Possessed by the Entire Molecule)

Monomer	Stretch	Bend	Stretch–Bend	Torsion	Non-1,4 VDW	1,4 VDW	Charge/Charge	Charge/Dipole	Dipole/Dipole	Total Energy (kcal/mol)
ACBK^a^	–	–	–	–	–	–	–	–	–	–
ACBK_na_	2.676	409.706	–1.270	94.647	–3.162	22.684	51.732	0.549	44.976	622.538
ACR176	2.940	276.848	–0.809	62.086	–4.310	23.018	22.184	–0.774	27.935	409.118
ACR176_na_	2.157	274.632	–0.798	56.368	–1.056	24.990	22.046	–2.965	30.224	405.598
AlR	3.201	276.319	–0.757	60.899	–2.702	25.307	15.635	–3.660	27.735	401.976
AlR_na_	2.831	273.495	–0.659	60.818	0.074	25.852	15.452	–1.274	29.842	406.431
AYR	1.119	7.006	0.164	–11.160	2.917	19.088	–2.897	–1.023	0.803	16.018
AYR_na_	0.986	5.544	0.185	–11.160	2.878	20.531	–2.951	–1.068	0.170	15.115
AzRS	4.320	416.259	–1.175	91.834	0.090	28.248	54.258	–6.326	43.049	630.559
AzRS_na_	3.839	410.597	–1.026	94.177	0.331	28.643	51.189	–5.981	45.477	627.247
CFB	3.502	280.623	–0.700	53.443	1.660	27.847	16.057	0.197	28.782	411.411
CFB_na_	3.152	277.939	–0.615	52.470	1.564	28.754	16.050	–0.921	29.972	408.364
DB15	7.888	567.216	–1.349	112.317	–0.224	50.401	104.234	–10.702	58.406	888.188
DB15_na_	6.707	560.635	–1.247	107.546	2.443	51.737	103.299	–5.489	60.013	885.644
DB71	7.907	563.865	–1.128	106.246	2.151	61.089	106.120	0.763	60.087	907.100
DB71_na_	7.366	562.442	–1.108	103.942	3.087	62.446	106.217	–4.358	59.261	899.294
EBT	2.719	142.903	–0.322	16.671	1.425	28.484	–1.763	–15.629	15.773	190.261
EBT_na_	2.226	141.607	–0.312	10.338	2.020	30.778	–2.037	–3.476	16.034	197.179
EB^a^	–	–	–	–	–	–	–	–	–	–
EB_na_	6.638	557.416	–1.425	122.193	1.676	43.580	122.450	2.692	61.451	916.671
HNB	4.327	416.898	–1.234	92.896	–6.419	27.012	54.189	–4.317	43.926	627.279
HNB_na_	3.839	410.560	–1.026	94.204	0.340	28.644	51.202	–6.000	45.478	627.243
PaRe	3.043	143.066	0.155	–24.193	–0.680	28.522	0.000	–4.604	14.708	160.018
PaRe_na_	2.241	138.483	0.304	–25.600	3.846	30.751	0.000	–4.458	15.590	161.336
Pon	3.303	279.530	–0.642	56.771	–0.439	27.922	30.496	–17.455	30.826	410.312
Pon_na_	3.279	275.652	–0.688	57.945	–0.118	28.826	27.824	–8.974	29.763	413.509
PR	2.854	282.649	–0.533	74.563	–2.612	30.565	28.504	–26.899	33.196	422.287
PR_na_	2.490	281.012	–0.462	63.829	–2.099	31.437	27.706	–23.150	34.449	415.212
Sudan III	1.940	10.683	0.194	–24.293	1.413	31.447	–	–	–2.369	19.015
Sudan III_na_	1.628	8.343	0.248	–24.500	1.308	32.539	–	–	–1.018	18.548
SY	2.448	272.307	–0.763	62.122	–0.966	20.995	15.106	–1.251	28.534	398.532
SY_na_	2.148	270.085	–0.713	61.777	–1.001	22.053	14.949	–1.668	29.648	397.279
TRT	2.687	285.396	–0.874	66.743	–1.702	19.098	55.205	–1.964	35.228	459.817
TRT_na_	2.415	284.164	–0.877	66.782	–0.354	19.569	55.476	–0.828	33.481	459.829
TrB	6.858	565.540	–1.478	126.835	5.778	45.070	118.638	–28.808	60.555	898.987
TrB_na_	5.839	554.017	–1.463	111.689	–0.136	48.189	112.867	3.717	59.214	893.931
Y2G	2.646	282.357	–0.244	30.524	0.240	21.901	–	–8.217	37.743	366.950
Y2G_na_	2.389	280.919	–0.244	26.918	2.437	21.911	–	–7.767	36.091	362.654

a MM2 terms could not be calculated for ACBK and EB due to high VDW interactions.

When examining the total energy parameter in Table 5, it can be observed that the steric energies of the natural hydroxyl-containing forms of the monomers are generally higher. Lower steric hindrance is preferred for straightforward electropolymerization. However, by focusing on other energy types, it is evident that the 1,4 VDW interactions show lower energy values for all monomers with hydroxyl groups compared to their hydroxyl-free forms. When analyzing stretch–bend values, a similar trend is observed, except for the Pon and TRT monomers, where the hydroxyl-free forms exhibit slightly lower energy values. However, the percentage differences are minimal compared to their hydroxyl-containing forms. In the case of the Y2G monomer, the energy values are equal. A similar pattern is generally observed for the non-1,4 VDW energy type, with only the AYR and CFB monomers showing slightly higher energy values than their hydroxyl-free forms. The only exception is the TrB molecule, where the hydroxyl-containing monomer exhibits significantly higher energy. This is likely due to long-range interactions between the hydroxyl and methyl groups at distances exceeding three atoms. Nevertheless, when examining all three energy types (stretch–bend, non-1,4 VDW, and 1,4 VDW) are considered collectively, it is generally observed that the nonhydroxylated form of the monomer exhibits a higher steric energy. Therefore, when evaluations are based solely on these three types of energies and their combined effects, the lower steric energy values obtained for the hydroxyl-containing monomers can be regarded as a strong indicator of enhanced electrocatalytic activity.

In conclusion, when conducting electropolymerization experiments with hydroxyl-functionalized azo dyes, these parameters can be pre-examined to make highly accurate predictions, ultimately saving time and resources.

## 6 Conclusion and Future Perspectives

This study reviewed the production and applications of electrochemical sensor platforms based on the electropolymerization of hydroxyl-functionalized azo dyes. Possible polymer structures and electrode reaction mechanisms of the related analytes were proposed for the studies where no information was provided about these characteristics. The mechanisms by which these polymers sense specific analytes were thoroughly explained with detailed insights. The rationale behind the electrocatalytic effects was clarified through comprehensive calculations using the extended Hückel and MM2 methods.

This study is expected to accelerate research on sensor applications involving the electropolymerization of hydroxyl-functionalized azo dyes. More importantly, researchers can perform extended Hückel and MM2 calculations, as outlined in this review, before conducting any experiments for electropolymerization. This might allow for highly accurate predictions and the efficient use of time and resources. Alternatively, researchers may refer to this review and directly utilize the electropolymerization of hydroxyl-functionalized azo dyes for various research applications.

In addition, future studies could explore the integration of hydroxyl-functionalized azo polymers into flexible or miniaturized sensing platforms. Their tunable redox behavior and surface chemistry make them promising candidates for smart or wearable electrochemical sensor systems, particularly where real-time and on-site monitoring are required.

## Supplementary Material



## Notes

Electrochemical redox processes of all analytes detected by hydroxyl-functionalized azo-dye polymers (PDF)
