# Supplementary material for: Marvelous Characteristics of Hydroxyl-Functionalized Azo-Dye Polymers for Electrocatalytic Sensing: An Informative Review
Source: ACS Polym Au. 2025 Nov 24;6(1):61–85. doi: 10.1021/acspolymersau.5c00145 (PMC12903508; doi:10.1021/acspolymersau.5c00145)
Supplement: Supplementary file 1 [file lg5c00145_si_001.pdf]

Marvelous characteristics of hydroxyl-functionalized azo-dye polymers for electrocatalytic sensing: An informative review

Lokman Liv*

*Electrochemistry Laboratory, Chemistry Group, The Scientific and Technological Research Council of Turkey, National Metrology Institute, (TUBITAK UME), 41470, Gebze, Kocaeli, Turkey.

E-mail: lokman.liv@tubitak.gov.tr

Contents **Page**

1. Electrocatalytic sensing mechanism of hydroxyl-functionalized azo-dye polymers.............2

1. Electrocatalytic sensing mechanism of hydroxyl-functionalized azo-dye polymers

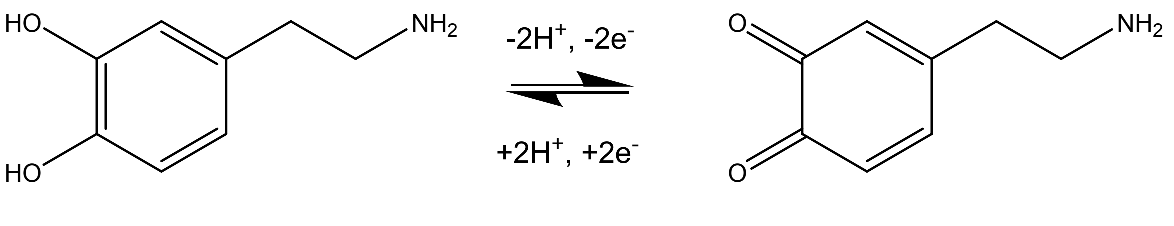

Dopamine

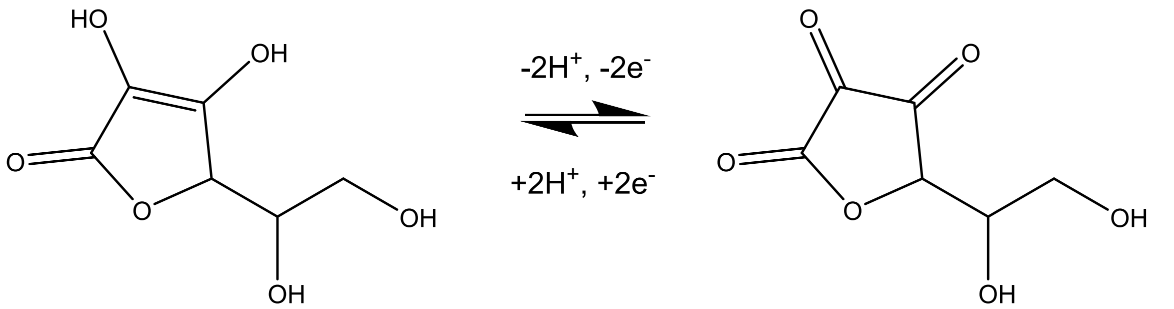

Ascorbiacid

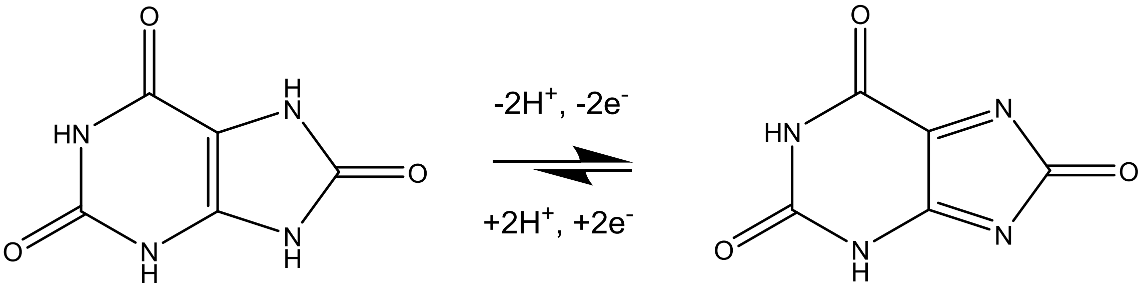

Uricacid

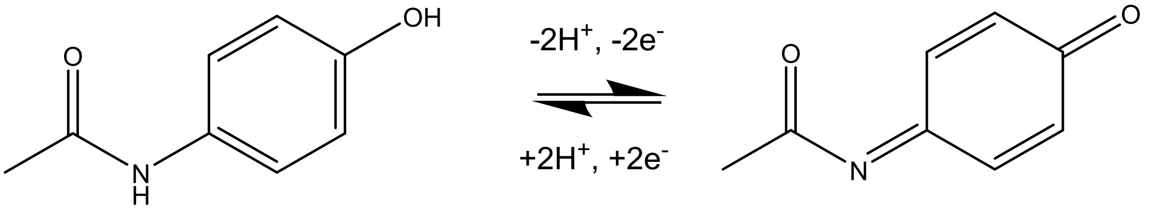

Acetaminophen

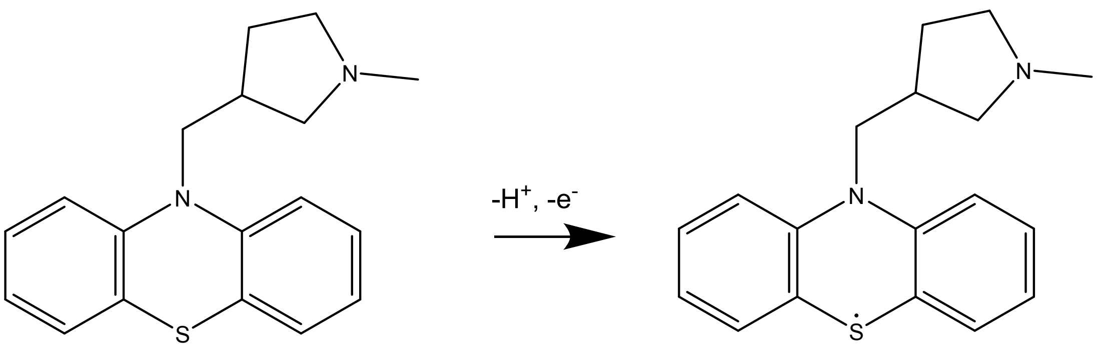

Methdilazine

$$N_2H_4 \longrightarrow N_2 + 4e^- + 4H^+$$

Hydrazine

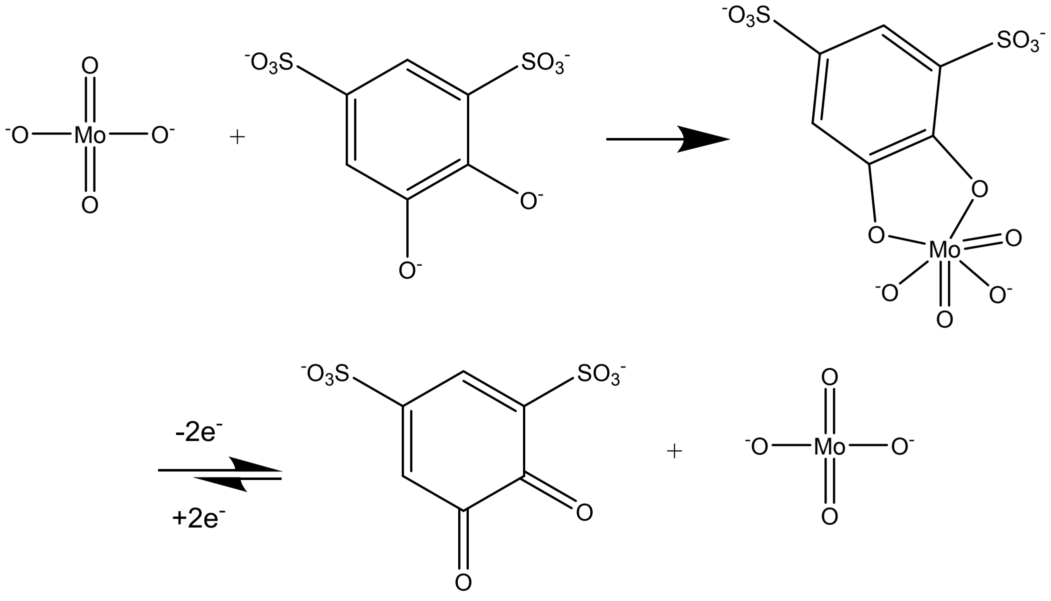

Molybdenum-Tiron

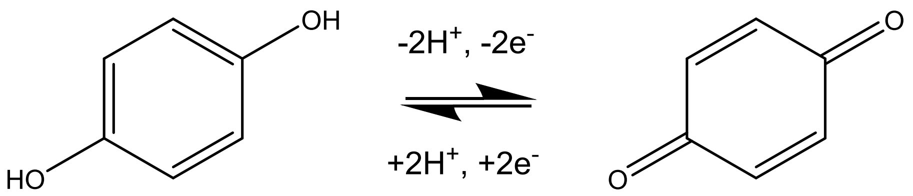

Hydroquinone

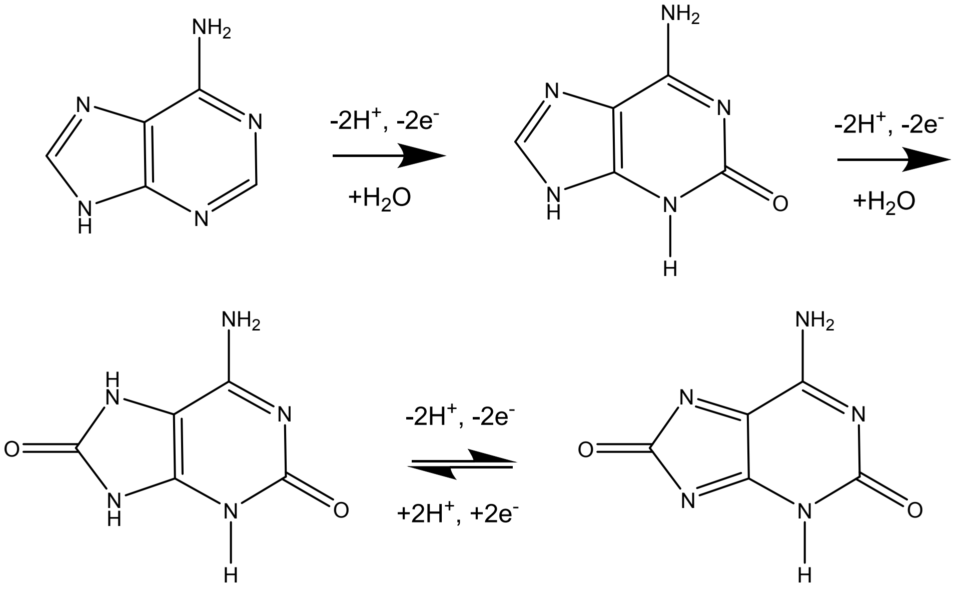

Adenine

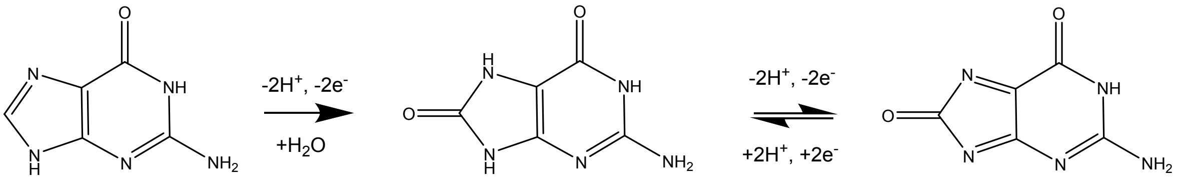

Guanine

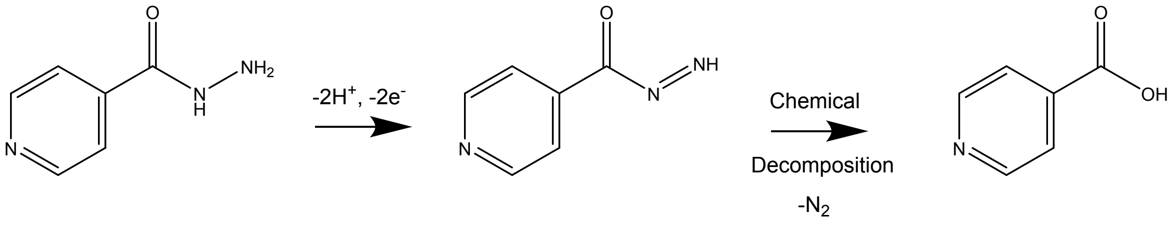

Isoniazid

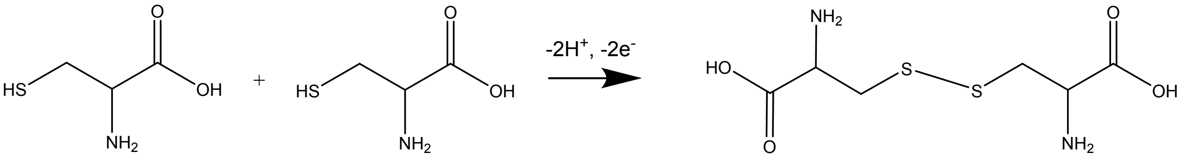

L-cysteine

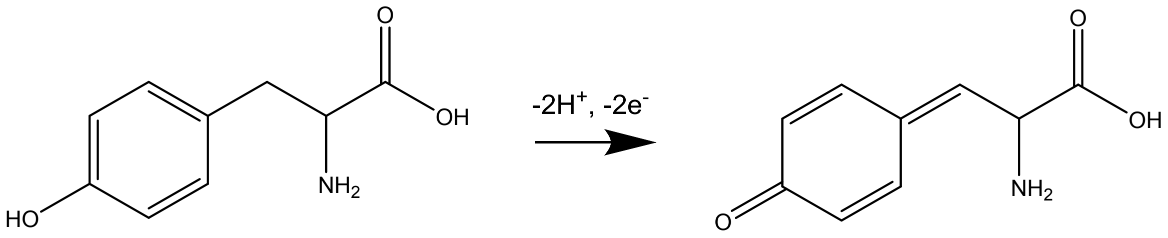

L-tyrosine

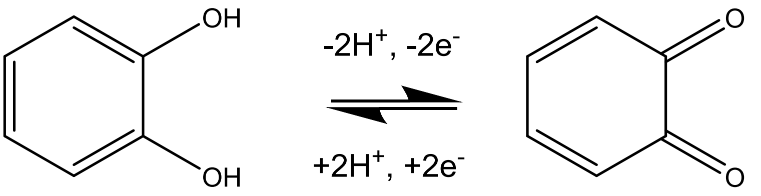

Catechol

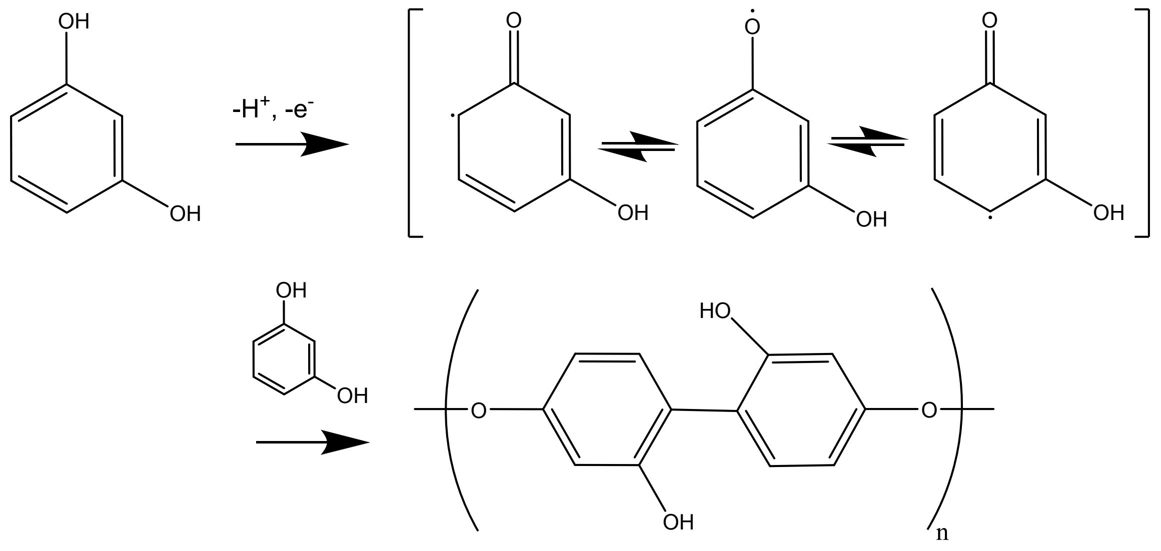

Resorcinol

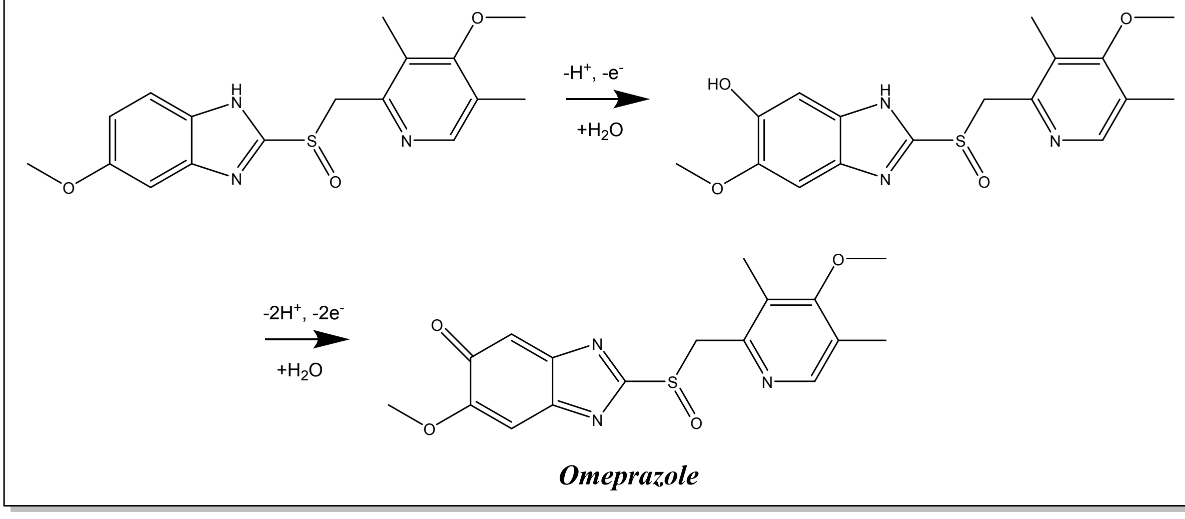

Omeprazole

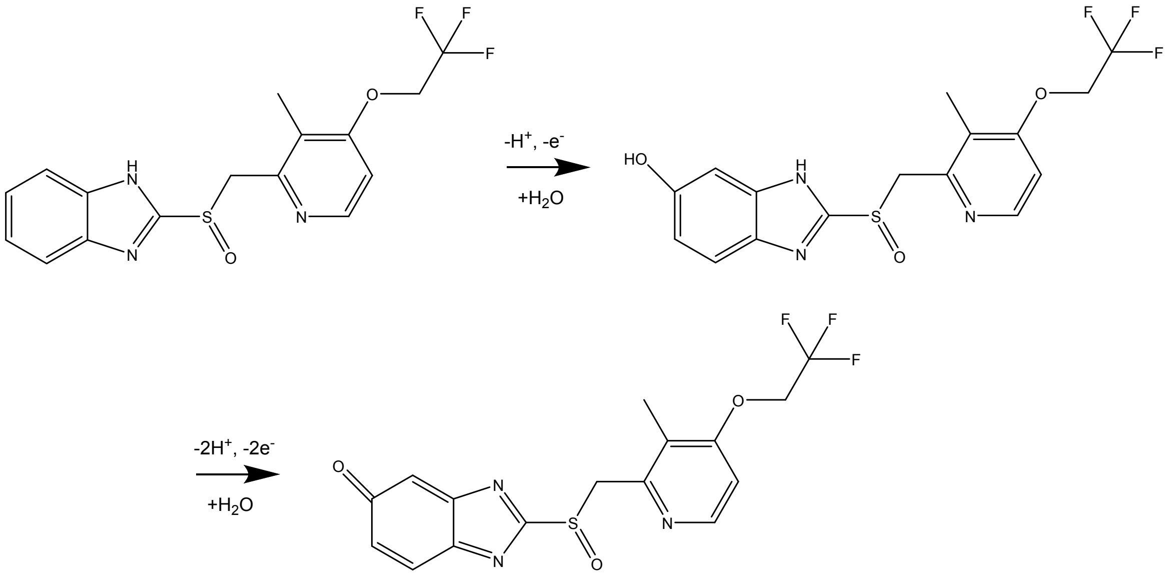

Lansoprazole

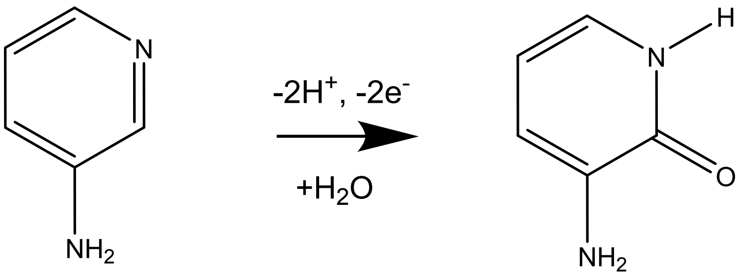

3-aminopyridine

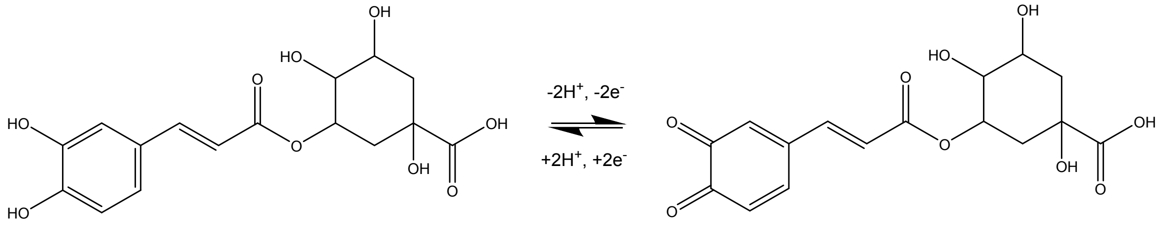

Chlorogeniacid

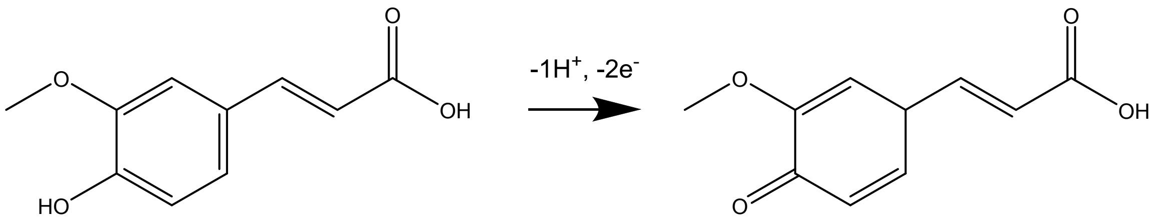

Feruliacid

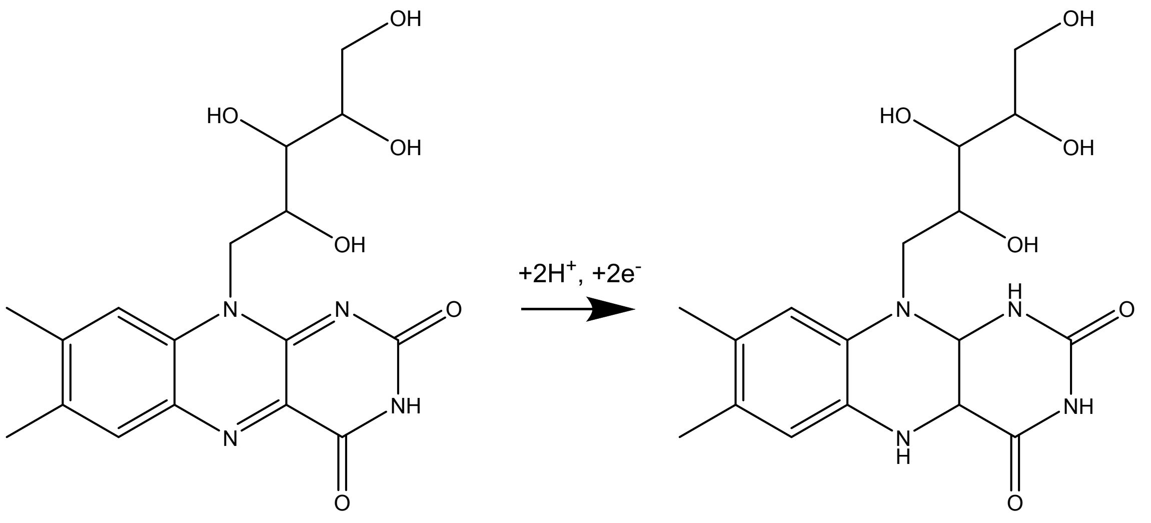

Riboflavin

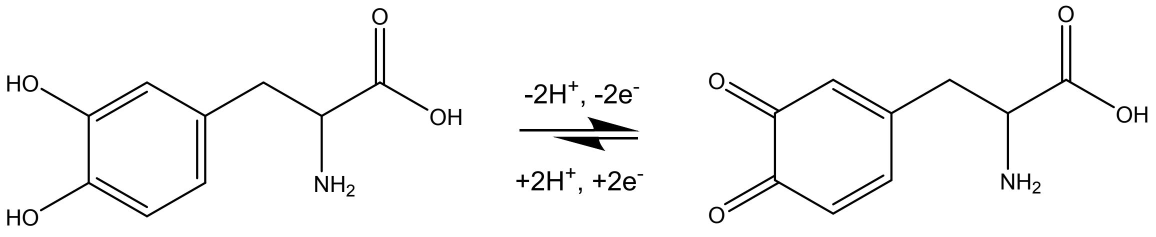

Levodopa

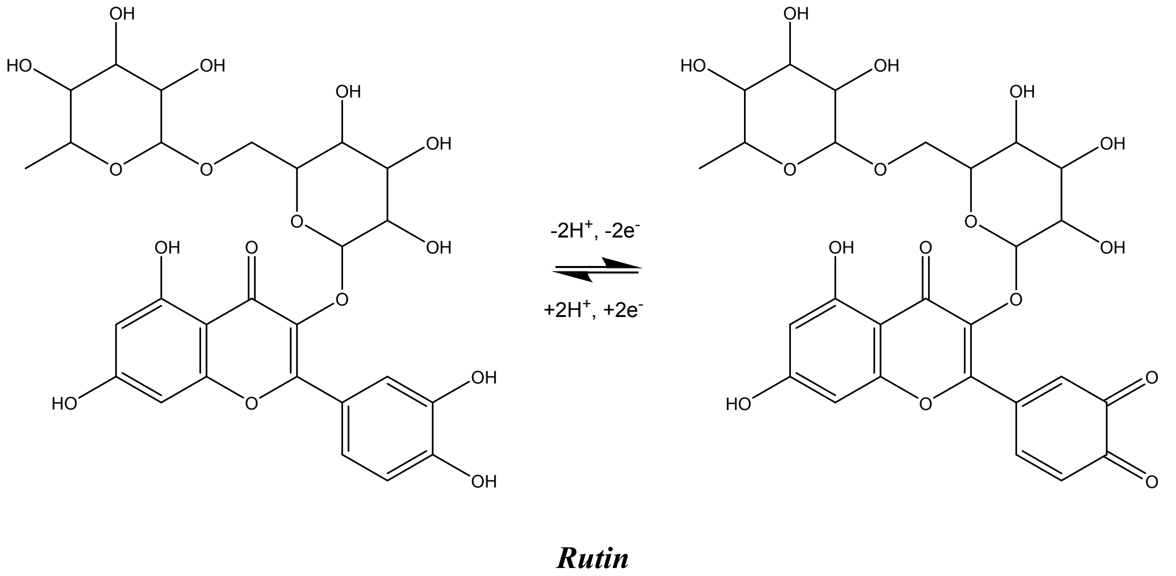

Rutin

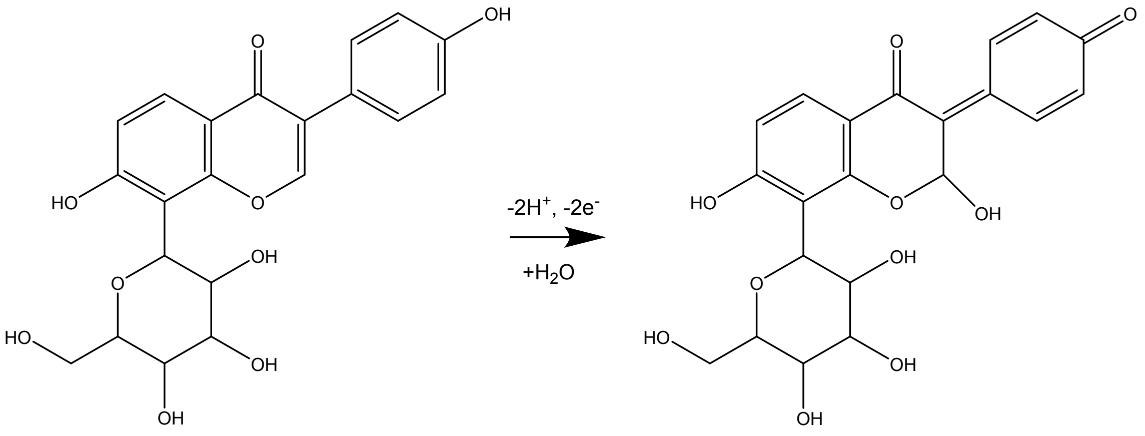

Puerarin

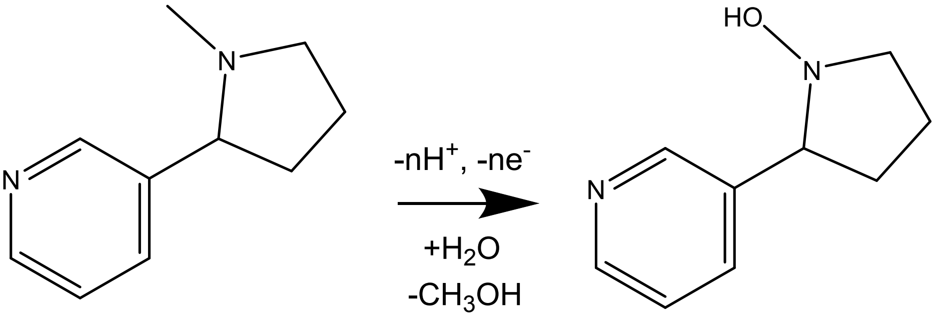

Nicotine

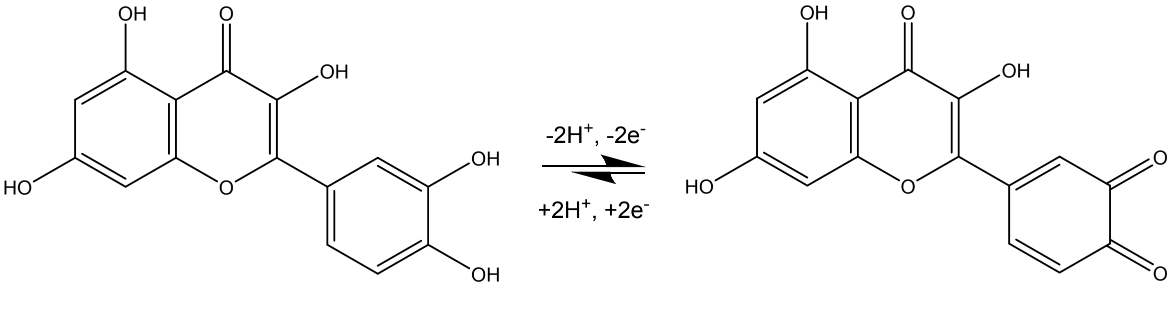

Quercetin

$$NO_3^- + 2e^- \longrightarrow NO_2^- + 2OH^-$$

$$NO_2^- + 4e^- \longrightarrow NH_2OH + 5OH^-$$

Nitrate&Nitrite

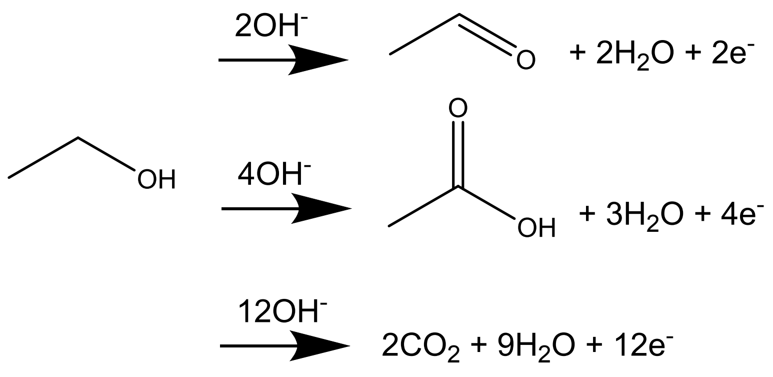

Ethanol

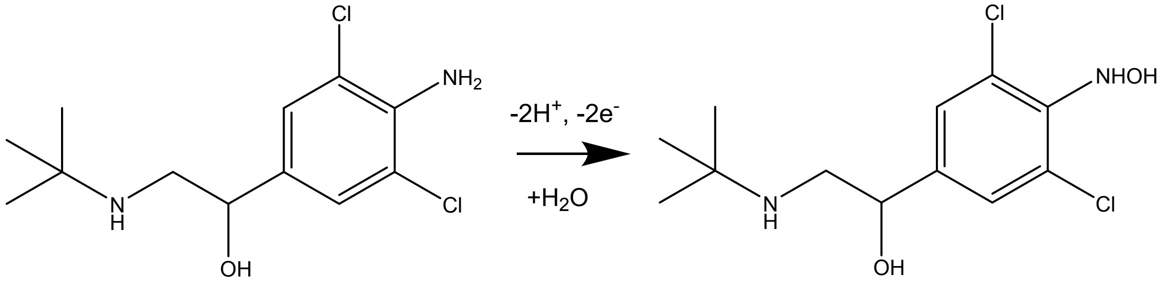

Clenbuterol

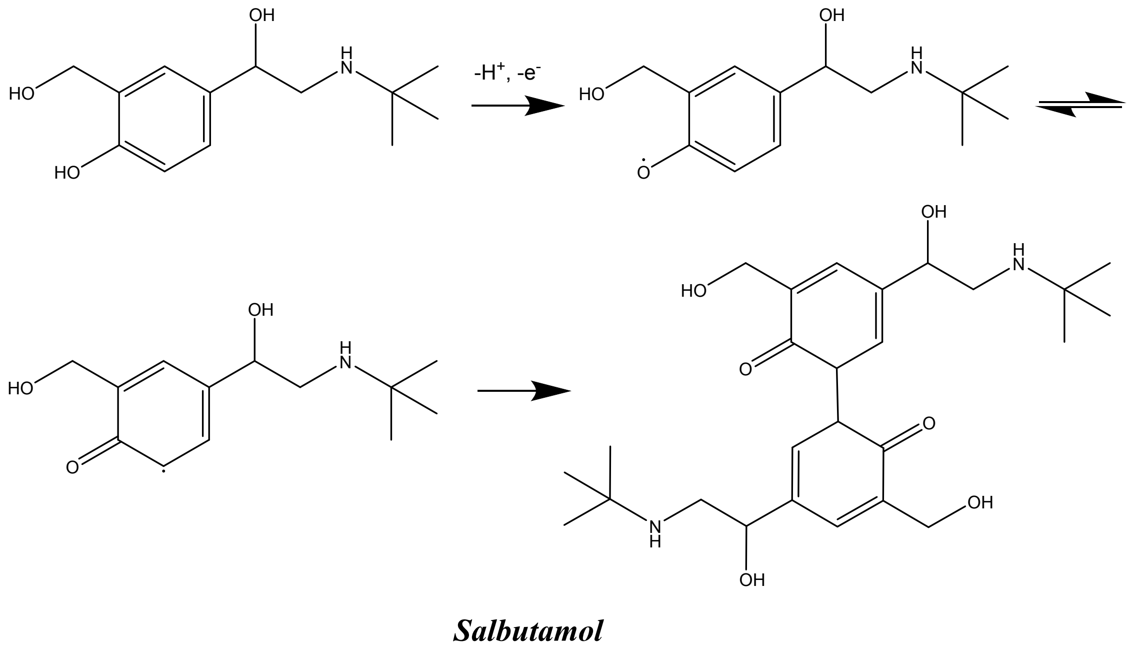

Salbutamol

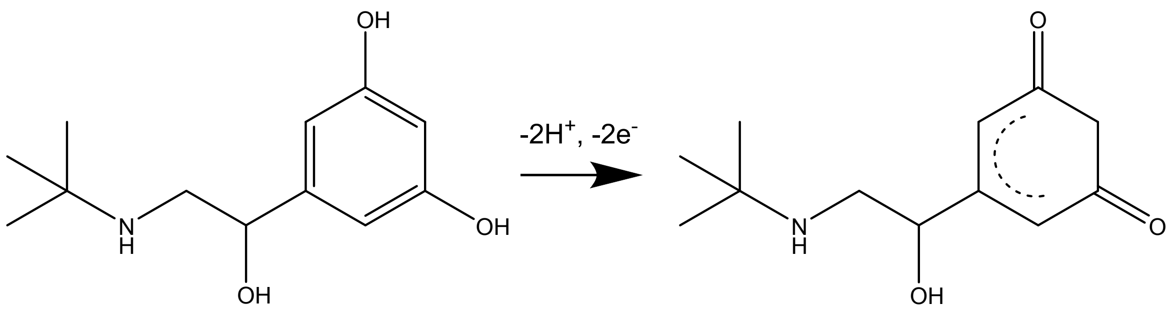

Terbutaline

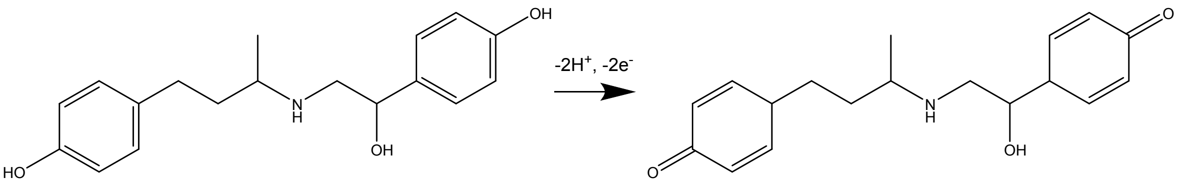

Ractopamine

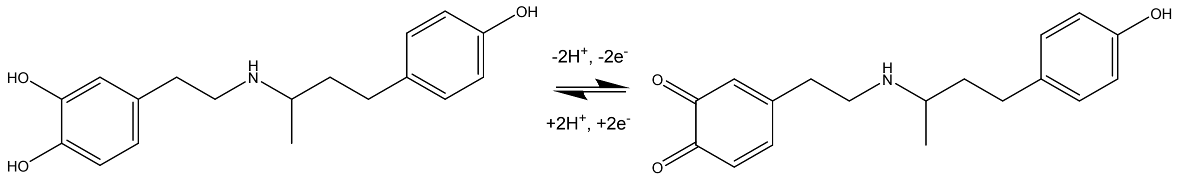

Dobutamine

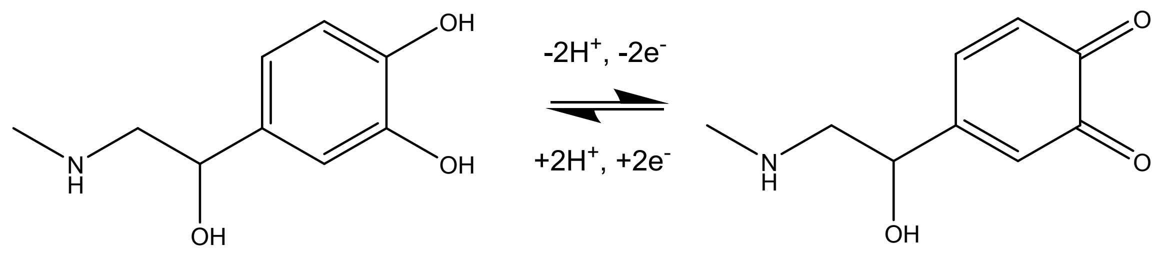

Adrenaline

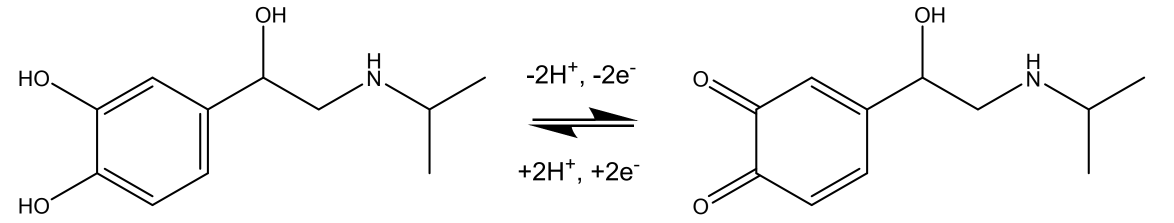

Isoprenaline

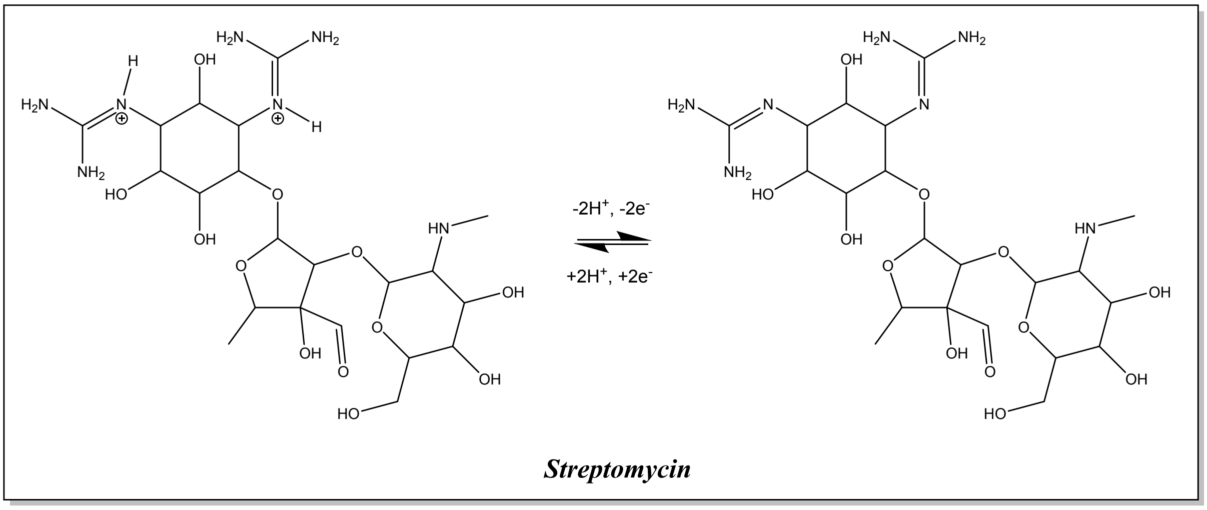

Fig. S1. Electrochemical redox processes of all analytes detected by hydroxyl-functionalized azo-dye polymers.
